# Bioactive Natural Compounds in Triple-Negative Breast Cancer: Molecular Targets and Therapeutic Perspectives

**DOI:** 10.3390/ph19040550

**Published:** 2026-03-30

**Authors:** Emilia Jiménez-Flores, Claudia Reytor-González, Dolores Jima Gavilanes, Cesar Carrillo, Raquel Horowitz, Jenny Carola Cárdenas Carrera, Gabriele Davide Bigoni-Ordóñez, Daniel Simancas-Racines

**Affiliations:** 1 Facultad de Ciencias de la Salud y Bienestar Humano, Universidad Tecnológica Indoamérica, Ambato 180150, Ecuador; emii300712@gmail.com; 2 Escuela de Medicina, Universidad Espíritu Santo, Samborondón 0901952, Ecuador; doloresjima78@hotmail.com; 3 Facultad de Medicina Veterinaria y Zootecnia, Universidad Agraria del Ecuador, Guayaquil 090104, Ecuador; cccarrillo@uagraria.edu.ec; 4 Department of Medicine, Geriatrics Division, Montefiore Medical Center, Bronx, NY 10467, USA; rahorowitz@montefiore.org; 5 Carrera de Laboratorio Clínico, Facultad de Ciencias Médicas, Universidad de Cuenca, Cuenca 010107, Ecuador

**Keywords:** triple-negative breast cancer, natural products, breast cancer, therapeutic strategies, novel molecular pathways, healthcare, polyphenols, terpenoid compounds, flavonoids, alkaloids, microbial products

## Abstract

Triple-negative breast cancer represents one of the most aggressive and therapeutically challenging subtypes of breast malignancies, characterized by marked biological heterogeneity, rapid progression, and limited targeted treatment options. Conventional therapies are frequently constrained by drug resistance, systemic toxicity, and high rates of recurrence. In this context, natural products have gained increasing attention as multifunctional agents capable of modulating several hallmarks of triple-negative breast cancer. Bioactive compounds, including polyphenols, terpenoids, alkaloids, and marine-derived molecules, exhibit pleiotropic antitumor effects by interfering with key oncogenic pathways. Importantly, these compounds have demonstrated the ability to counteract major mechanisms of therapeutic resistance, modulate the tumor immune microenvironment, and enhance the efficacy of standard chemotherapy and immunotherapy. Advances in drug delivery strategies, such as nanoparticle-based systems and tumor-targeted formulations, together with patient-specific molecular profiling, further expand the potential of these agents within personalized treatment approaches. This narrative review critically examines the role of natural compounds in targeting the hallmarks of triple-negative breast cancer and their potential synergistic use to improve therapeutic efficacy while reducing treatment-related toxicity. Overall, the integration of natural product-based strategies into precision oncology frameworks may offer more effective, less toxic, and individualized therapeutic options for this aggressive breast cancer subtype.

## 1. Introduction

Breast cancer remains the most frequently diagnosed malignancy among women worldwide and represents a major public health challenge. According to the Global Cancer Observatory (GLOBOCAN), approximately 2.3 million new cases and 670,000 breast cancer-related deaths were reported globally in 2022, with incidence continuing to rise in many regions [1]. Although advances in screening and treatment have improved survival in high-income regions, breast cancer continues to be the leading cause of cancer-related mortality among women, particularly in settings with limited access to early diagnosis and comprehensive care [2].

Pronounced geographic, ethnic, and socioeconomic disparities further shape disease burden, reflecting differences in genetic susceptibility, healthcare infrastructure, and social determinants of health [3,4]. In the United States, Black women experience significantly higher mortality despite lower incidence, while several Asian regions have reported rapidly increasing incidence rates [5,6]. These disparities are rooted in a multifactorial etiology involving genetics, reproduction, lifestyle, and environment. Established risk factors include early menarche, late first childbirth, lack of breastfeeding, oral contraceptive use, high-fat diets, alcohol, smoking, and high breast density. Genetic susceptibility, particularly *BRCA1/2* mutations, remains a significant risk determinant [7].

Beyond epidemiological trends, breast cancer is characterized by remarkable molecular and clinical heterogeneity, which profoundly influences prognosis and therapeutic response. Age- and population-specific differences in tumor biology have been described, with younger women in several Asian populations showing higher incidence rates, and women of African ancestry more frequently developing aggressive molecular subtypes, particularly triple-negative breast cancer (TNBC) [8]. Genomic analyses have identified population-specific mutational landscapes, including differential alterations in tumor protein p53 (TP53) and phosphatidylinositol-4,5-bisphosphate 3-kinase catalytic subunit alpha (PIK3CA), underscoring the need for biologically informed and population-adapted therapeutic strategies [9]. Although male breast cancer represents less than 1% of cases, it displays distinct biomarker profiles and poorer clinical outcomes, further highlighting the complexity of breast cancer biology [10,11]. Key risk factors for male breast cancer include advanced age, obesity, diabetes, Klinefelter’s syndrome, and occupational exposures to high heat or carcinogens like exhaust fumes [12]. Genetic predisposition is particularly pronounced, with 5–10% of cases attributed to inherited mutations, most frequently in *BRCA2*. Clinically, male breast cancer often presents as a painless, palpable subareolar mass [13].

TNBC accounts for approximately 10–25% of all breast cancer cases and is defined by the absence of estrogen receptor (ER), progesterone receptor (PR), and human epidermal growth factor receptor 2 (HER2) expression, which precludes the use of endocrine or HER2-targeted therapies and contributes to poor clinical outcomes [14]. TNBC has a partially distinct etiological profile. Genetic susceptibility, especially *BRCA1* mutations, plays a vital role [7]. Reproductive factors like early menarche, a lack of breastfeeding, and oral contraceptive use are implicated, while longer breastfeeding is protective. The higher prevalence in women of African ancestry points to a complex interplay of genetic and socioeconomic factors [15], and is often presented with basal-like tumors characterized by high histological grade, early recurrence, and metastatic propensity [16]. Recurrence typically occurs within the first three years after diagnosis and is frequently associated with chemoresistance and distant metastasis [17]. Five-year overall survival remains low, particularly in metastatic disease, where median survival rarely exceeds 15 months [18,19].

At the molecular level, TNBC is characterized by extensive pathway dysregulation, including aberrant activation of the phosphoinositide 3-kinase/protein kinase B/mechanistic target of rapamycin (PI3K/AKT/mTOR) and mitogen-activated protein kinase (MAPK) signaling axes, epithelial–mesenchymal transition (EMT)-associated programs, and frequent alterations in the breast cancer susceptibility gene (BRCA) [20]. These molecular alterations drive tumor aggressiveness, immune evasion, and therapeutic resistance, while simultaneously creating multiple actionable vulnerabilities that may be selectively targeted through multi-pathway modulation. Early-relapsing TNBC, characterized by intrinsic resistance to chemotherapy and immune checkpoint inhibitors, represents a particularly aggressive clinical phenotype with poor prognosis [21,22].

In this context, increasing interest has focused on natural products as sources of bioactive molecules capable of modulating multiple oncogenic pathways simultaneously. More than 60% of currently approved anticancer drugs are derived wholly or partially from natural sources, highlighting their longstanding relevance in oncology [23]. Diverse classes of phytochemicals—including polyphenols, alkaloids, terpenoids, and marine-derived compounds—have demonstrated antiproliferative, pro-apoptotic, anti-inflammatory, and anti-metastatic effects in TNBC models [24,25]. Natural compounds such as curcumin, resveratrol, and epigallocatechin-3-gallate (EGCG) have been shown to modulate key signaling pathways, remodel the tumor immune microenvironment, and enhance sensitivity to chemotherapy and immunotherapy, positioning them as promising candidates for combination-based therapeutic strategies [26,27].

Advances in network pharmacology, molecular docking, and nanotechnology-based delivery systems, such as nanoparticle (NP)-based formulations, have further strengthened the translational rationale for these agents by improving bioavailability, tumor targeting, and therapeutic efficacy [23,28,29,30].

Despite this expanding literature, most existing reviews primarily catalog individual compounds or signaling pathways without articulating a unifying conceptual framework integrating TNBC molecular heterogeneity, adaptive resistance mechanisms, and immune–metabolic interactions. In particular, the polypharmacological nature of natural products is often described as an inherent property rather than as a strategically exploitable therapeutic principle. A critical unresolved gap therefore remains in defining how multitarget bioactive compounds can be rationally aligned with TNBC molecular signatures and resistance phenotypes to inform precision-oriented combination strategies and enhance clinical relevance. The aim of this review is to propose an integrative, hallmark-oriented, and resistance-aware framework that links bioactive natural compounds with TNBC molecular signatures, therapeutic vulnerabilities, and emerging translational strategies, highlighting their potential role in rational combination approaches for this aggressive breast cancer subtype.

## 2. Methods

This narrative review considered publications from database inception through January 2025. Literature searches were conducted in PubMed/MEDLINE, Cochrane Library, and Google Scholar using terms including “natural products,” “breast cancer,” “therapeutic strategies,” “novel molecular pathways,” “polyphenols,” “terpenoid compounds,” “flavonoids,” “alkaloids,” “microbial products,” and “triple-negative breast cancer.” Eligible articles included original research, systematic reviews, meta-analyses, narrative reviews, and clinical guidelines addressing the effects of natural compounds on breast cancer outcomes. Studies examining molecular mechanisms, therapeutic strategies, or translational applications of natural products—particularly in triple-negative breast cancer—were included, along with clinical trials evaluating natural compound-based interventions. Selected articles were analyzed to synthesize evidence on the therapeutic potential, molecular pathways, and clinical relevance of natural products in breast cancer management.

## 3. Cancer Hallmarks in TNBC: A Molecular Snapshot

### 3.1. Overview of Hallmarks

Tumors, like normal tissues, require a continuous supply of nutrients and oxygen and the efficient removal of metabolic waste to sustain growth. In cancer, these demands are met through the formation of tumor-associated neovasculature driven by pathological angiogenesis. Angiogenesis involves the sprouting of new vessels from pre-existing vasculature; in adults, angiogenesis is typically a tightly regulated and transient process, occurring primarily during wound healing and the menstrual cycle. In contrast, tumor progression is characterized by the persistent activation of an “angiogenic switch,” which continuously drives neovascularization to support uncontrolled proliferative growth [31].

This angiogenic switch is regulated by the imbalance between pro-angiogenic and anti-angiogenic signals. Pro-angiogenic mediators such as vascular endothelial growth factor A (VEGF-A) and fibroblast growth factors (FGFs) are upregulated in response to hypoxia, oncogenic signaling, and extracellular matrix (ECM) remodeling, whereas endogenous inhibitors, including thrombospondin-1, angiostatin, and endostatin, are concurrently suppressed [32,33]. As a consequence, tumor-associated blood vessels formed under chronic angiogenic stimulation exhibit profound structural and functional abnormalities; these include irregular branching, distorted morphology, erratic blood flow, increased permeability, and dysregulated endothelial cell proliferation and apoptosis, collectively contributing to tumor survival and resistance to cell death [34,35]. Importantly, angiogenesis is activated early during tumorigenesis, sustaining proliferation and enabling malignant progression [36].

Tumor cells further evade growth suppressive signals and resist apoptosis through alterations in cell–cell adhesion, most notably a loss of E-cadherin, which promotes cell detachment, invasion, and metastatic dissemination [37,38]. The invasion–metastasis cascade involves local invasion, dissemination, and colonization of distant organs [39]. Cancer cells often exploit EMT to acquire enhanced motility, invasiveness, extracellular matrix–degrading capacity, and resistance to apoptosis, a process driven by transcription factors such as Snail, Slug, Twist, and zinc finger E-box-binding homeobox 1/2 (ZEB1/2) [40]. EMT is increasingly recognized as a dynamic and reversible process, with cells undergoing partial EMT states or mesenchymal–epithelial transition (MET) in response to microenvironmental cues, underscoring the plasticity of malignant cells [41].

The tumor microenvironment (TME) plays a critical role in facilitating invasion and metastasis. Stromal components, including mesenchymal stem cells and tumor-associated macrophages, promote tumor dissemination through reciprocal signaling, secretion of growth factors, and production of matrix-degrading enzymes [42]. In addition to single-cell migration, tumor cells can disseminate collectively or adopt amoeboid modes of movement, while inflammatory cells at the invasive front remodel the ECM, reducing the dependence on EMT-associated proteolytic activity [36].

Metastatic progression requires not only successful dissemination but also efficient colonization of secondary sites. Micrometastases frequently enter a dormant state, maintained by insufficient angiogenic support or inhibitory signals from the local microenvironment [43]. Successful metastatic outgrowth depends on the acquisition of adaptive traits that support sustained proliferation, evasion of growth suppressors, induction of angiogenesis, and maintenance of replicative immortality [36]. Circulating tumor cells are also capable of reseeding the primary tumor, thereby modifying its gene expression profile and phenotypic characteristics, highlighting the bidirectional and interconnected nature of invasion, metastasis, and angiogenesis [44,45,46]. In TNBC, these hallmark programs are frequently co-activated and tightly interconnected, contributing to aggressive clinical behavior, early relapse, and the rapid emergence of therapeutic resistance.

In addition to these classical hallmarks, TNBC progression is strongly influenced by metabolic and immune-related adaptations that shape tumor evolution. A key aspect of this is metabolic reprogramming, where cancer cells alter their energy production pathways to fuel uncontrolled growth. TNBC cells prominently exhibit the Warburg effect (aerobic glycolysis), favoring glycolysis for energy production even in the presence of oxygen, which supports rapid proliferation and provides biosynthetic precursors [47]. This is often coupled with altered oxidative phosphorylation and enhanced glutamine utilization to meet biosynthetic demands and maintain redox homeostasis [48]. These metabolic shifts not only sustain tumor growth but also influence the TME by generating an acidic, nutrient-depleted niche through the accumulation of metabolites like lactate, which can impair antitumor immune cell function. Concurrently, TNBC tumors actively remodel the immune landscape to evade destruction. This immune modulation involves the secretion of immunosuppressive cytokines that recruit and expand regulatory T cells and myeloid-derived suppressor cells [49]. A central mechanism of immune evasion is the upregulation of immune checkpoint molecules, most notably programmed death-ligand 1 (PD-L1), on tumor cells, which engages with programmed cell death protein 1 (PD-1) on cytotoxic T cells to suppress their activity and induce exhaustion [50]. Together, this metabolic rewiring and immune modulation represent key enabling characteristics that facilitate tumor progression, metastatic dissemination, and therapeutic resistance in TNBC.

Collectively, these coordinated capabilities exemplify how tumors acquire and integrate multiple cancer hallmarks—including sustained proliferative signaling, evasion of growth suppression, resistance to cell death, angiogenesis, invasion, and metastasis—to drive malignant progression [36]. The key molecular biomarkers associated with these hallmarks are summarized in Table 1.

### 3.2. Emphasis on Key Altered Pathways in TNBC

#### 3.2.1. Sustained Proliferation

Sustained proliferative signaling in TNBC is largely driven epidermal growth factor receptor (EGFR) overexpression and hyperactivation, as well as frequent alterations in the PI3K/AKT/mTOR pathway [60]. EGFR promotes malignant expansion through activation of the RAS/MAPK and Signal Transducer and Activator of Transcription 3 (STAT3) pathways, thereby enhancing cell cycle progression, survival, and tumor growth. EGFR signaling is further amplified by USP8 overexpression and loss of PTPN12, a tumor suppressor phosphatase that normally restrains multiple oncogenic kinases, including EGFR [61].

Therapeutic targeting of EGFR in TNBC has yielded limited clinical benefit. This is largely due to persistent activation of downstream signaling pathways. Constitutive PI3K/AKT/mTOR signaling represents a major mechanism of intrinsic resistance to EGFR-directed therapies, with PI3K or PTEN mutations frequently underlying primary treatment failure [62].

Class I PI3Ks play a central role in TNBC tumorigenesis, and PIK3CA mutations, identified in a subset of TNBC cases, increase PIP3 production, thereby promoting sustained proliferation and suppressing apoptosis [55,63,64]. Activated AKT isoforms further regulate growth, invasion, and survival, with AKT3 being preferentially enriched and functionally relevant in TNBC [65]. mTORC1 and mTORC2 sustain biosynthetic activity, cytoskeletal remodeling, and full AKT activation through phosphorylation, reinforcing proliferative and survival signaling [66,67].

Collectively, these findings support a model in which aberrant EGFR and PI3K/AKT/mTOR signaling cooperatively sustain uncontrolled proliferation in TNBC, highlighting the therapeutic rationale for combined EGFR–mTOR or multi-node pathway inhibition to achieve synergistic antitumor effects and overcome resistance mechanisms [68].

#### 3.2.2. Evasion of Apoptosis

Over the past two decades, the B-cell lymphoma 2 (BCL-2) protein family has been systematically classified based on the presence of conserved BCL-2 homology (BH) domains and is functionally divided into anti-apoptotic members (including BCL-2, BCL-XL, and MCL-1), pro-apoptotic effector proteins (BAX and BAK), and BH3-only pro-apoptotic regulators (such as BAD, BID, BIM, and PUMA) [58]. Anti-apoptotic BCL-2 family members, characterized by BH1–BH4 domains and a C-terminal transmembrane region, preserve mitochondrial outer membrane integrity. They do so by sequestering BH3-only proteins and preventing BAX/BAK oligomerization, thereby inhibiting cytochrome c release [69,70,71].

In addition to regulating mitochondrial apoptosis, anti-apoptotic BCL-2 proteins influence intracellular Ca^2+^ homeostasis, redox balance, and caspase activation, suppressing caspase-9, caspase-3, caspase-6, and caspase-7-mediated apoptotic signaling [69,70,71]. Their overexpression in malignant cells confers resistance to multiple cellular stresses, positioning BCL-2 family inhibition as a rational therapeutic strategy in cancer [72]. In breast cancer, dysregulation of BCL-2 family proteins has been associated with impaired apoptosis, altered cell cycle control, and enhanced tumor cell survival, while context-dependent reductions in BCL-2 expression have also been implicated in tumor initiation and progression [73,74].

Apoptotic evasion in TNBC is further reinforced by frequent TP53 mutations, which compromise DNA damage sensing, cell cycle arrest, and pro-apoptotic transcriptional programs. Mutant TP53 interferes with p63 and p73 signaling and cooperates with AKT-mediated repression of BCL-2-modifying factor, thereby promoting cell survival and resistance to apoptosis [75,76]. Collectively, alterations in BCL-2 family signaling and TP53-dependent apoptotic pathways constitute central mechanisms of apoptotic escape in TNBC, profoundly influencing tumor aggressiveness and responsiveness to cytotoxic chemotherapy [77].

#### 3.2.3. Angiogenesis

TNBC is characterized by intratumoral hypoxia, which stabilizes hypoxia-inducible factor (HIF)-α proteins and enhances their transcriptional activity [68]. HIF-1α overexpression occurs early during breast carcinogenesis, including ductal carcinoma in situ, and is associated with higher tumor grade, increased invasiveness, and poor prognosis, particularly in necrotic and oxygen-deprived tumor regions [57]. In TNBC, recurrent molecular alterations—such as TP53 loss, PTEN deficiency, EGFR overexpression, and hyperactivation of the PI3K/AKT/mTOR or MAPK pathways—further enhance HIF-α expression and stability even under normoxic conditions, thereby uncoupling angiogenic signaling from oxygen availability [78,79]. HIF-2α, whose expression is partially regulated by forkhead box A1 (FOXA1), also contributes to breast cancer progression and angiogenic signaling, reinforcing hypoxia-driven tumor adaptation [80,81].

The TNBC TME is intensely hypoxic resulting from high metabolic oxygen demand combined with structurally and functionally inadequate vasculature [82]. Chronic inflammation further exacerbates hypoxia through sustained production of reactive oxygen species (ROS) and nitric oxide, which stabilize HIF-1α and prolong its transcriptional activity [83]. Concomitantly, HIF-1α orchestrates metabolic adaptation by promoting glycolytic reprogramming, inducing glucose transporter 1 and multiple rate-limiting glycolytic enzymes, thereby supporting tumor survival under oxygen-limited conditions [84,85].

HIF-1α activation in TNBC robustly induces angiogenesis. Through transcriptional upregulation of VEGF, hepatocyte growth factor (HGF), vascular cell adhesion molecule 1 (VCAM1), and VEGF receptors, HIF-1α directly promotes neovascular formation and endothelial cell activation [86]. Additional pro-angiogenic inputs—including ROS-dependent induction of brain-derived neurotrophic factor (BDNF) and HIF-1α/TAZ–mediated co-activation of VEGF and insulin-like growth factor 2 (IGF2)—further amplify angiogenic signaling networks in TNBC [87]. Moreover, stromal and immune components of the TME contribute complementary angiogenic cues, with mast cell-derived VEGF, interleukin-8 (IL-8), and transforming growth factor beta 1 (TGFβ1) reinforcing pathological vascular remodeling and sustained tumor perfusion [88].

#### 3.2.4. Immune Evasion and Inflammation

In TNBC, immune escape and chronic inflammation are largely driven by aberrant activation of nuclear factor kappa B (NF-κB) and overexpression of PD-L1, promoting tumor progression and immune suppression. NF-κB is frequently hyperactivated in TNBC, supporting tumor growth, angiogenesis, survival, and resistance to cell death [89]. NF-κB activation is initiated through cytokine-dependent inhibitor of kappa B kinase (IKK) signaling, as well as ROS-mediated degradation of inhibitors of kappa B alpha (IκBα) under hypoxic conditions, which concurrently enhances HIF-1α transcriptional activity and induces cyclooxygenase-2 (COX-2) expression [83,90].

Functionally, NF-κB orchestrates extensive inflammatory and immune-related transcriptional programs, regulating cytokine and chemokine production, T-cell recruitment and polarization, and cell survival pathways. It also promotes EMT, angiogenesis, genomic instability, and immunosuppressive remodeling of the TME [91,92]. NF-κB dysregulation is further exacerbated by loss of NOD-like receptor family pyrin domain containing 12 (NLRP12), which leads to enhanced TNBC proliferation and metastatic dissemination, as well as by overexpression of receptor-interacting protein kinase 2 (RIPK2), a key upstream activator of NF-κB-driven inflammatory and metastatic signaling [93,94].

PD-L1 overexpression constitutes a complementary immune evasion mechanism, impairing the function of T lymphocytes, natural killer (NK) cells, dendritic cells (DCs), and macrophages, thereby facilitating immune escape [49]. PD-L1 expression is induced by interferon gamma (IFNγ)-mediated Janus kinase/signal transducer and activator of transcription (JAK/STAT)–interferon regulatory factor 1 (IRF1) signaling, as well as by pro-inflammatory cytokines and oncogenic pathways, including EGFR, MYC proto-oncogene (MYC), and Yes-associated protein/transcriptional co-activator with PDZ-binding motif (YAP/TAZ) [50,95]. Across breast cancer subtypes—and particularly in TNBC—PD-L1 expression plays a central role in shaping the immunosuppressive landscape and determining responsiveness to PD-1/PD-L1 immune checkpoint blockade [96,97,98].

#### 3.2.5. Invasion and Metastasis

Matrix metalloproteinases (MMPs) are zinc-dependent endopeptidases that degrade components of the extracellular matrix (ECM) and play a central role in cancer progression, including tumor growth, invasion, and metastatic dissemination [99,100]. Key MMPs, such as MMP-2, MMP-9, and MMP-1, not only facilitate ECM remodeling and tumor cell migration but also modulate immune responses within the TME, enhancing invasion [101]. MMP-1, predominantly produced by cancer-associated fibroblasts, has been shown to stimulate breast cancer cell proliferation, whereas MMP14 (MT1-MMP) is critically required for efficient metastatic spread in TNBC [102].

Transforming growth factor beta (TGF-β) signaling exerts a context-dependent role in cancer, acting as a tumor suppressor during early tumorigenesis but promoting tumor progression at advanced stages by inducing EMT. In TNBC, TGF-β-driven EMT enhances migratory and invasive capacity, contributes to chemotherapy resistance, and is associated with poor clinical outcomes [103]. EMT is orchestrated by transcription factors including Snail, Slug, Twist, and ZEB1/2, which are directly activated downstream of TGF-β signaling cascades [104]. Loss-of-function mutations in TP53 further accelerate EMT progression, in part through upregulation and stabilization of Snail, thereby amplifying metastatic potential [105].

In TNBC, EMT is closely linked to aggressive disease behavior and the acquisition of stem cell–like properties, which sustain tumor initiation, plasticity, and therapeutic resistance [56]. Post-translational regulation, particularly ubiquitination, plays a critical role in controlling EMT dynamics by modulating the stability of key transcription factors such as Snail. The E3 ubiquitin ligase membrane-associated ring-CH-type finger 2 (MARCH2) targets Snail for proteasomal degradation, thereby suppressing EMT, invasion, and metastatic progression, highlighting ubiquitin-mediated regulation as a promising therapeutic vulnerability in TNBC [106].

Beyond EMT-driven local invasion, TNBC exhibits a pronounced propensity for brain metastasis, a clinically significant challenge associated with poor prognosis [107]. Successful colonization of the brain requires circulating tumor cells to traverse the blood–brain barrier (BBB) through protease-mediated junctional disruption, transcytosis, and dynamic interactions with brain endothelial cells, astrocytes, and microglia, which collectively support survival, metabolic adaptation, immune evasion, and outgrowth within the central nervous system microenvironment [108,109]. Reciprocal tumor–brain signaling further reinforces brain-specific metastatic progression, involving adaptive rewiring of oncogenic pathways such as sustained PI3K/AKT/mTOR activation and cytokine-driven JAK/STAT signaling, which also contribute to therapeutic resistance [110]. The brain metastatic niche is additionally characterized by relative immune suppression and altered immune checkpoint expression, including PD-L1, underscoring the unique biology of TNBC brain metastases and highlighting BBB penetration, tumor–brain crosstalk, and central nervous system immune context as critical considerations for therapeutic strategies [110].

#### 3.2.6. Metabolic Rewiring

**Figure 1:**
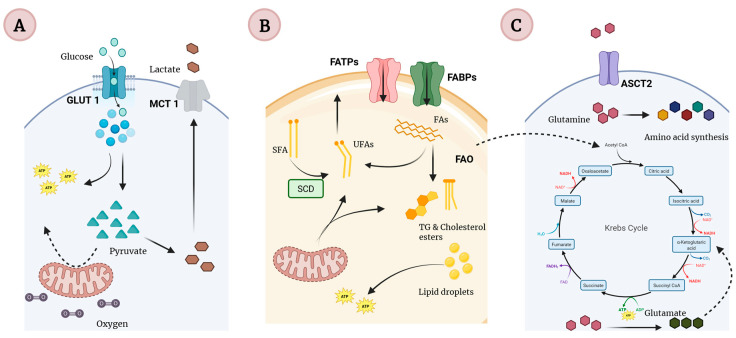
Metabolic reprogramming in TNBC cells. (**A**) Aerobic glycolysis (Warburg effect) characterized by increased glucose uptake and lactate production, supporting biosynthesis and tumor–microenvironment acidification. (**B**) Fatty acid metabolism reprogramming, including enhanced uptake, de novo synthesis, desaturation, storage, and mitochondrial β-oxidation, which sustain membrane remodeling, energy balance, and therapeutic resistance. (**C**) Glutamine metabolism, marked by increased glutamine uptake and glutaminolysis, replenishing tricarboxylic acid cycle intermediates and supporting redox homeostasis and rapid proliferation. Collectively, these interconnected metabolic programs promote TNBC growth, plasticity, and survival [118,119,120]. Abbreviations: GLUT1: Glucose transporter 1; MCT1: Monocarboxylate transporter 1; ATP: Adenosine triphosphate; UFAs: Unsaturated fatty acids; TG: triglyceride; SFA: Saturated fatty acid; FATPs: Fatty acid transport proteins; FABPs: Fatty acid-binding proteins; SCD: Stearoyl-CoA desaturase; FAO: Fatty acid oxidation; ASCT2: Alanine, serine, cysteine-preferring transporter 2; CO_2_: Carbon dioxide; NAD: Nicotinamide adenine dinucleotide; NADH: Nicotinamide adenine dinucleotide (reduced form); FAD: Flavin adenine dinucleotide; FADH_2_: Flavin adenine dinucleotide (reduced form); H_2_O: Water; Acetil-CoA: Acetyl–Coenzyme A.

Metabolic reprogramming enables cancer cells to adapt to environmental stress, sustain rapid proliferation, and evade therapy through coordinated alterations in glycolysis, oxidative phosphorylation (OXPHOS), and lipid and amino acid metabolism (Figure 1). These metabolic programs are regulated by key transcriptional and signaling hubs, including HIF-1, MYC, TP53, peroxisome proliferator-activated receptors (PPARs), ER, and sterol regulatory element-binding proteins (SREBPs) [111]. A central feature of cancer-associated metabolic reprogramming is the Warburg effect, whereby tumor cells preferentially rely on aerobic glycolysis to generate ATP and biosynthetic intermediates, even under normoxic conditions [57,112].

TNBC displays a higher degree of metabolic plasticity than predicted by the classical Warburg model, exhibiting both increased and decreased OXPHOS activity across tumor subsets and frequently relying on a hybrid glycolytic–OXPHOS metabolic state that supports survival and growth across diverse and fluctuating metastatic niches [113]. This metabolic flexibility is tightly coupled to glutamine metabolism, which serves as a critical carbon and nitrogen source by sustaining the tricarboxylic acid cycle, macromolecule biosynthesis, and glutathione production through glutaminolysis [114].

TNBC cells frequently overexpress glutamine transporters, including alanine–serine–cysteine transporter 2 and L-type amino acid transporter 1 (LAT1), thereby enhancing glutamine uptake and activating mTORC1-dependent anabolic signaling. Metabolomic analyses consistently reveal reduced intracellular glutamine levels and elevated glutamate concentrations, reflecting accelerated glutaminolytic flux [115]. High glutamine consumption by TNBC cells not only fuels tumor growth but also depletes nutrients within the TME. This impairs immune cell function, promoting immune evasion, and increasing tumor vulnerability to glutamine-targeted therapeutic strategies [49,116].

Notably, the majority of glutamine-derived carbon enters the tricarboxylic acid cycle without complete oxidation, following a “single-pass” glutaminolysis pattern analogous to lactate overflow in glycolysis, which underscores the heightened glutamine dependency of TNBC compared with other breast cancer subtypes [117].

### 3.3. Clinical Translation: Early Molecular Diagnosis and Therapeutic Targeting

Early diagnosis and molecular characterization are critical for improving outcomes in patients with TNBC. Given the high risk of early relapse within the first 2–3 years after diagnosis, timely identification of targetable alterations is particularly urgent. Advances in molecular diagnostics, particularly next-generation sequencing, have enabled comprehensive profiling of tumor genomes and transcriptomes, facilitating the detection of clinically relevant alterations including germline and somatic mutations in *BRCA1/2*, *TP53*, *PIK3CA*, and other genes involved in DNA repair and oncogenic signaling pathways [121].

Beyond next-generation sequencing, a growing arsenal of molecular diagnostic approaches has expanded the toolkit for TNBC detection and characterization. Liquid biopsy techniques have emerged as particularly valuable, enabling non-invasive analysis of tumor-derived materials from blood, saliva, or urine [122]. These approaches include circulating tumor DNA analysis, which can detect minimal residual disease and identify relapse months before clinical or radiological evidence [123]. Digital droplet PCR and BEAMing technology offer ultrasensitive detection of known mutations at allele frequencies as low as 0.001%, making them ideal for monitoring treatment response and early recurrence [124]. Circulating tumor cells can be isolated and characterized at the single-cell level, with recent studies identifying novel surface markers such as AHNAK2, CAVIN1, ODR4, and TRIML2 that enhance TNBC circulating tumor cells detection specifically [125]. RNA-based approaches, including microRNA (miRNA) profiling—particularly miR-21—and long non-coding RNA analysis, have shown promise as diagnostic and prognostic indicators [126]. Extracellular vesicles and exosomes isolated from biofluids carry tumor-specific cargo including proteins, mRNAs, and miRNAs, offering another window into tumor biology [122]. DNA methylation markers detected in liquid biopsies have demonstrated promising diagnostic accuracy for TNBC, with tumor-specific methylation patterns enabling both early detection and disease monitoring [126]. Multi-omics strategies integrating genomics, transcriptomics, proteomics, and metabolomics are increasingly applied to uncover novel non-invasive biomarkers and deepen understanding of TNBC heterogeneity [122]. The comparative performance of various screening and diagnostic approaches available for TNBC detection and monitoring is summarized in Table 2.

Such insights not only enhance understanding of TNBC heterogeneity but also support the development of precision oncology strategies, allowing for the selection of targeted therapies such as Poly (ADP-Ribose) Polymerase (PARP) inhibitors or immune checkpoint inhibitors in appropriate patient populations. Consequently, integrating early molecular diagnostics into clinical practice represents an important step toward improving TNBC management.

### 3.4. Current Therapeutic Landscape and Limitations in TNBC

Despite advances in understanding TNBC biology, current treatment options remain limited and are often associated with significant challenges. For decades, cytotoxic chemotherapy has been the mainstay of treatment for TNBC, typically involving regimens based on taxanes (paclitaxel, docetaxel), anthracyclines (doxorubicin, epirubicin), and platinum agents (carboplatin, cisplatin) [38]. While TNBC often shows initial chemosensitivity, with pathological complete response rates reaching approximately 46–52% in the neoadjuvant setting, a substantial proportion of patients exhibit residual disease, which correlates with a high risk of recurrence and poor survival outcomes [133]. Furthermore, chemotherapy is associated with considerable toxicity, including myelosuppression, neuropathy, and anthracycline-induced cardiotoxicity, which can compromise patient quality of life and limit long-term use [134]. The development of chemoresistance, frequently driven by processes such as EMT, remains a major obstacle to durable disease control [38]. In recent years, the treatment landscape has expanded with the incorporation of targeted and immunotherapeutic agents. For patients with germline *BRCA1/2* mutations, PARP inhibitors (olaparib, talazoparib) have demonstrated efficacy by exploiting DNA repair deficiencies, though acquired resistance to these agents is an emerging clinical challenge [135]. The introduction of immune checkpoint inhibitors has represented a paradigm shift, with pembrolizumab (anti-PD-1) in combination with chemotherapy now established as a standard of care for both early-stage (regardless of PD-L1 status) and PD-L1-positive metastatic TNBC. Similarly, atezolizumab (anti-PD-L1) combined with nab-paclitaxel is approved for first-line treatment of PD-L1-positive metastatic disease [136]. Despite these advances, only a subset of patients derives durable benefit from immunotherapy, and immune-related adverse events require careful management. Most recently, antibody–drug conjugates such as sacituzumab govitecan, targeting TROP-2, have shown remarkable efficacy in pretreated metastatic TNBC and are being investigated in earlier lines, with emerging data suggesting potential to redefine first-line standards [137]. Surgery and radiotherapy remain essential components of locoregional control, particularly following neoadjuvant therapy, but do not address systemic dissemination [38]. Given the persistent challenges of treatment resistance, heterogeneous therapeutic response, and the lack of benefit for a significant proportion of patients, there remains an urgent need for novel therapeutic strategies with distinct mechanisms of action, leading to the exploration of natural product-based therapies.

## 4. Plant-Derived Natural Compounds Targeting TNBC Hallmarks

**Figure 2:**
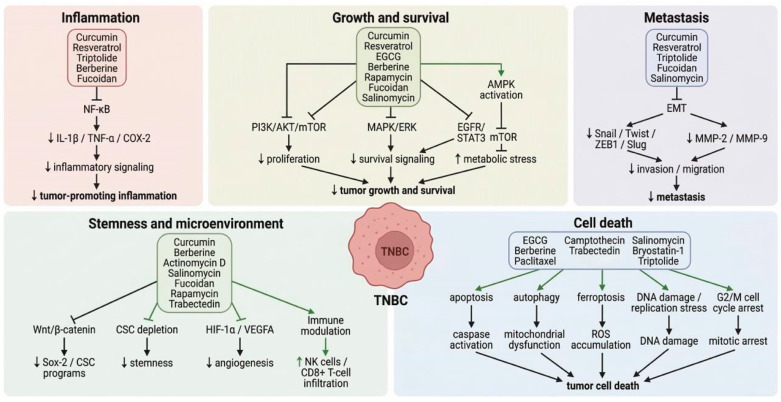
Multitargeted mechanisms of natural compounds in cancer hallmarks. This summarized representation organizes key natural compounds according to their primary impact on the biological hallmarks of cancer. It illustrates how individual agents, such as Curcumin, Resveratrol, and Salinomycin, can simultaneously interfere with multiple signaling pathways to suppress inflammation, inhibit growth and survival, block metastasis, deplete cancer stem cells, modulate the tumor microenvironment, and ultimately induce various forms of cell death [139,140,141,142,143,144,145,146,147,148,149,150,151]. Abbreviations: NF-κB: nuclear factor kappa-light-chain-enhancer of activated B cells; IL-1β: interleukin-1 beta; TNF-α: tumor necrosis factor-alpha; COX-2: cyclooxygenase-2; PI3K: phosphoinositide 3-kinase; AKT: protein kinase B; mTOR: mammalian target of rapamycin; MAPK: mitogen-activated protein kinase; ERK: extracellular signal-regulated kinase; EGFR: epidermal growth factor receptor; STAT3: signal transducer and activator of transcription 3; AMPK: AMP-activated protein kinase; EMT: epithelial–mesenchymal transition; Snail: snail family transcriptional repressor 1; Twist: twist family transcription factor; ZEB1: zinc finger E-box-binding homeobox 1; Slug: snail family transcriptional repressor 2; MMP-2: matrix metalloproteinase-2; MMP-9: matrix metalloproteinase-9; Wnt: wingless-related integration site signaling pathway; β-catenin: beta-catenin; CSC: cancer stem cell; Sox-2: SRY-box transcription factor 2; HIF-1α: hypoxia-inducible factor 1-alpha; VEGFA: vascular endothelial growth factor A; NK cells: natural killer cells; CD8+ T-cells: cluster of differentiation 8-positive T lymphocytes; ROS: reactive oxygen species; EGCG: epigallocatechin-3-gallate; TNBC: triple-negative breast cancer. ⊣ = inhibition; → = downstream consequence or activation; ↓ = decrease; ↑ = increase.

Growing evidence indicates that natural compounds can target key hallmarks of TNBC [138]. As summarized in Figure 2, these compounds simultaneously interfere with multiple interconnected pathways—including inflammation, proliferation, metastasis, stemness, and cell death—to suppress tumor progression. However, it is important to clarify that TNBC is primarily driven by genetic mutations, which natural products cannot directly correct. Instead, these compounds exert their effects by modulating the downstream signaling pathways and biological processes disrupted by such mutations, thereby suppressing malignant phenotypes and enhancing therapeutic responsiveness. Despite chemotherapy and immunotherapy being standard treatments, their efficacy is often limited [25]. Bioactive phytochemicals have shown anti-TNBC activity and may act synergistically with chemotherapeutic agents to enhance efficacy and overcome resistance (Table 3).

### 4.1. Polyphenols

Integrating metabolic and molecular perspectives highlights the therapeutic relevance of bioactive compounds such as curcumin, resveratrol, and epigallocatechin-3-gallate (EGCG), which modulate key metabolic, inflammatory, and epigenetic pathways driving tumor development. These agents interfere with aerobic glycolysis, glutamine utilization, lipid biosynthesis, and pro-survival signaling cascades, consequently disrupting the metabolic adaptability that supports cancer cell growth, immune escape, and metastatic potential [173,174].

Among these molecules, curcumin, a polyphenolic compound extracted from *Curcuma longa*, exhibits broad-spectrum anticancer effects by targeting inflammation, metabolism, and oncogenic signaling. It attenuates chronic inflammation by blocking NF-κB activation, reducing IL-1β, TNF-α, and COX-2 expression [26,175,176]. At the metabolic level, curcumin suppresses key glutamine transporters, consequently limiting the availability of amino acids required for tumor energy production and macromolecule synthesis [175]. These metabolic effects are accompanied by the inhibition of oncogenic signaling pathways, such as PI3K/AKT/mTOR, RAS, and Wnt/β-catenin, leading to reduced cellular proliferation, angiogenesis, and invasive capacity [177,178].

In breast cancer models, curcumin induces cell cycle arrest at the G2/M phase by increasing Glycogen Synthase Kinase 3-β activity and decreasing nuclear β-catenin accumulation, which in turn suppresses cyclin D1 expression [179]. Additionally, it inhibits EMT by downregulating transcription factors such as Snail, Twist, ZEB1, Slug, and vimentin, while promoting epithelial features and diminishing the expression of metastasis-associated proteins, including MMP-9, uPA, ICAM-1, and VEGF [142]. In TNBC, curcumin suppresses AXL/AKT-driven EMT and facilitates AMP-Activated Protein Kinase-mediated autophagic degradation of AKT, reducing migration and invasion [180,181,182]. Importantly, curcumin has also been shown to reverse chemoresistance, as evidenced by increased responsiveness to gemcitabine in resistant cholangiocarcinoma models, supporting its potential role as a chemosensitizing agent [183].

Resveratrol, a polyphenol found in pistachios, peanuts, bilberries, blueberries, and grapes, also demonstrates broad antitumor activity by modulating metabolic processes, survival pathways, and epigenetic mechanisms [184]. It decreases lactate production, thereby alleviating acidification of the TME and limiting both invasion and immune escape. In TNBC, resveratrol promotes mitochondrial dysfunction, ROS generation, lipid peroxidation, glutathione depletion, and ferroptosis by inhibiting the Extracellular Signal-Regulated Kinases (ERK1/2)–Serum/Glucocorticoid-Regulated Kinase 1–Neural Precursor Cell Expressed Developmentally Downregulated Protein 4-Like–Glutathione Peroxidase 4 signaling axis [185]. Furthermore, it suppresses EMT by facilitating β-TrCP-dependent degradation of Twist1 and counteracts EMT associated with AKT-targeted treatments [186].

Mechanistically, resveratrol inhibits NF-κB and PI3K/AKT signaling, downregulates anti-apoptotic proteins (Bcl-2, survivin, and XIAP), activates p53- and caspase-mediated apoptotis and impairs lipid raft signaling by reducing Fatty Acid Synthase, p-FAK, p-AKT, and p-ERK1/2 [187,188]. Additionally, it decreases glycolytic flux and ATP production via phosphoglycerate kinase-1 interaction and c-Myc suppression, while enhancing TNBC sensitivity to endocrine therapy and inhibiting hypoxia-driven metastasis through STAT3 acetylation reduction and tumor suppressor demethylation [189,190,191].

EGCG, the main catechin found in green tea, further reinforces these anticancer actions by targeting genomic instability, cellular proliferation, and survival pathways. It induces apoptosis stabilizes p53 function, and inhibits EGFR, MAPK, PI3K/AKT, STAT3, and Bcl-2 signaling, resulting in reduced proliferation and increased apoptosis [178,192,193]. It also suppresses fatty acid synthase activity, potentiates the cytotoxicity of standard chemotherapeutic agents, and, in combination with histone deacetylase inhibitors, markedly reduces TNBC migration and invasiveness, particularly under EMT conditions [194].

### 4.2. Terpenoids

Paclitaxel (PTX), found on the bark of Pacific Yew (*Taxus brevifolia*), exhibits strong anticancer activity in TNBC by stabilizing microtubules through binding to α- and β-tubulin subunits. This interaction disrupts mitotic spindle dynamics, resulting in chromosome segregation defects, G2/M cell cycle arrest, and activation of apoptotic pathways [195]. In addition, metronomic administration of low-dose PTX has been shown to increase its cytotoxic effects in TNBC, particularly when used alongside muscarinic M2 receptor agonists. This combination reduces the expression of chemoresistance- and aggressiveness-associated markers such as ATP-Binding Cassette Subfamily G Member 2 (ABCG2) and EGFR, while also decreasing tumor cell migration and angiogenic capacity [196].

In a similar context, triptolide—an active compound isolated from *Tripterygium wilfordii* Hook F—has demonstrated notable antitumor effects in TNBC by inhibiting cellular proliferation, migration, invasion, and vasculogenic mimicry. It promotes apoptosis, diminishes cancer stem-like characteristics, and facilitates lysosome-dependent degradation of the EMT regulator Twist 1. At the same time, it reduces Notch1 levels and suppresses NF-κB phosphorylation. These molecular changes lead to a downregulation of pro-metastatic and pro-angiogenic genes, including N-cadherin, VE-cadherin, and VEGFR2, thereby limiting the aforementioned processes [197]. Furthermore, triptolide activates c-Jun N-terminal Kinase signaling, resulting in increased expression of SNAI1, which represents a feedback mechanism linked to acquired resistance. This effect can be partially mitigated through SNAI 1 silencing, underscoring triptolide’s potential as a promising therapeutic candidate for TNBC [197].

### 4.3. Alkaloids

Natural alkaloids have gained attention as potential therapeutic agents for TNBC because of their ability to interfere with key molecular pathways implicated in tumor progression, such as PI3K/AKT/mTOR, STAT3, TGF-β, NF-κB, and p38 MAPK [198]. Within this group, berberine (BBR) and camptothecin (CPT) have demonstrated significant anticancer activity in experimental TNBC models. Mechanistically, the antitumor effects of these alkaloids converge on the regulation of cell survival, DNA integrity, and metastatic behavior.

BBR is an isoquinoline alkaloid extracted from *Cotridis rhizoma* and is known for its antimicrobial, anti-inflammatory, and antitumor properties [199]. In the context of TNBC, BBR suppresses tumor cell proliferation both in vitro and in vivo by triggering mitochondria-dependent apoptosis through the caspase-9/cytochrome c pathway and inducing cell cycle arrest at the G1, S, and G2/M checkpoints via regulation of the AKT/mTOR and PTEN/AKT/mTOR signaling axes [200,201]. Beyond its pro-apoptotic and antiproliferative effects, BBR influences autophagy through mechanisms involving AMP-Activated Protein Kinase, MAPK, Beclin-1/Bcl-2, and mTOR, while also modulating the Wnt/β-catenin and MAPK/ERK pathways. These mechanisms contribute to the suppression of metastatic potential, altering the expression of key molecular targets, including microRNAs (miRNAs) and p53 [202,203]. Notably, BBR has been shown to selectively target TNBC cells without exerting cytotoxic effects on non-tumorigenic breast epithelial cells [204].

The inhibition of the gene sirtuin 2 further leads to suppression of the NF-κB signaling pathway, which is frequently overactivated in TNBC, thereby reinforcing BBR’s antitumor effects [205]. Collectively, these findings highlight BBR as a multitarget modulator of TNBC hallmarks, with coordinated effects on proliferation, survival signaling, and metastatic dissemination [201].

Similarly, CPT, a naturally occurring alkaloid obtained from *Camptotheca acuminata*, exhibits strong cytotoxic properties across various cancer types, including TNBC [206]. In contrast to the pleiotropic signaling modulation observed with BBR, the primary mechanism of action of CPT involves the inhibition of DNA topoisomerase I, resulting in DNA damage by interfering with normal strand cleavage and re-ligation processes. This disruption ultimately hinders DNA replication and compromises cancer cell survival [207].

## 5. Non-Plant Natural Products with Anticancer Activity in TNBC

Marine and microbial-derived natural products represent potent therapeutic candidates in TNBC due to their capacity to modulate pathways that are insufficiently targeted by conventional therapies. Beyond cytotoxicity, many of these compounds exert unique, multitargeted mechanisms, including suppression of cancer stem cell (CSC) populations, reprogramming of oncogenic and epigenetic networks, and remodeling of tumor–immune interactions. These features directly address key drivers of TNBC progression—therapeutic resistance, immune evasion, and metastatic competence—highlighting the relevance of marine agents such as fucoidan, bryostatin-1, and trabectedin, together with microbial products including rapamycin, actinomycin D, and salinomycin, as promising components of advanced and combination-based therapeutic strategies [208].

### 5.1. Promising Marine Compounds

Marine-derived bioactive compounds represent a promising source of anticancer agents due to their ability to simultaneously interfere with tumor cell signaling, metastatic dissemination, DNA repair pathways, and immune regulation. Among these, fucoidan, bryostatin-1, and trabectedin have demonstrated significant therapeutic potential in breast cancer through complementary mechanisms involving tumor cell signaling, metastasis, DNA repair, and immune modulation [209].

Fucoidan is a sulfated, fucose-enriched polysaccharide that displays moderate growth-inhibitory effects in TNBC cells, while exerting pronounced anti-migratory and anti-invasive activities. These effects are largely attributed to inhibition of the MAPK and PI3K pathways, resulting in reduced transcriptional activity of AP-1 and NF-κB [210]. Fucoidan further modulates the PTEN/PI3K/AKT axis in a dose-dependent manner, decreasing PI3K/AKT phosphorylation and thereby weakening pro-survival signaling. Additionally, it significantly suppresses HIF-1α protein levels, limiting hypoxia-associated tumor adaptation [211].

Beyond upstream signaling inhibition, fucoidan translates these molecular effects into functional outcomes, promoting apoptotic cell death and disrupting cell cycle regulatory pathways. At lower concentrations, it has been reported to exhibit cytotoxic effects that may exceed those of doxorubicin. Fucoidan also modulates the TME by elevating IL-6 levels while reducing immunosuppressive and pro-metastatic mediators, such as TGF-β, particularly when used in combination with doxorubicin [212]. Moreover, it influences EMT by facilitating the interaction of Snail, Zeb, and Twist with the E-cadherin promoter and by regulating EMT-associated microRNAs, ultimately constraining metastatic potential [210].

Bryostatin-1, a macrolide compound isolated from the marine bryozoan *Bugula neritina*, functions as a powerful regulator of protein kinase C isoforms [149]. Upon binding to PKC, bryostatin-1 triggers enzyme activation via autophosphorylation and membrane translocation; sustained exposure subsequently promotes PKC ubiquitination and proteasomal degradation. Through these mechanisms, bryostatin-1 suppresses cellular proliferation, induces differentiation and apoptosis, inhibits MMP production, downregulates MDR1 expression, and modulates Bcl-2 and p53 signaling pathways [149].

Mechanistically, one key effect of bryostatin-1 is the induction of serine phosphorylation of the anti-apoptotic protein Bcl-2, resulting in its functional inhibition, although context-specific anti-apoptotic effects have also been described [213]. While its pro-apoptotic activity as a monotherapy is limited, bryostatin-1 significantly enhances the antitumor efficacy of chemotherapeutic agents such as gemcitabine and PTX. This synergistic interaction is mediated by increased caspase activation [214]. These findings have supported its clinical evaluation primarily in combination-based treatment strategies, given the modest outcomes observed with single-agent administration.

Trabectedin is another marine-derived alkaloid that exerts its antitumor effects by binding to the DNA minor groove and disrupting nucleotide excision repair mechanisms, ultimately leading to defective DNA repair and cancer cell death [211]. Tumors harboring BRCA2 deficiencies exhibit heightened sensitivity to trabectedin-induced apoptosis, underscoring its relevance for genetically stratified breast cancer subtypes. In clinical settings, trabectedin has demonstrated activity in patients with BRCA1/2-mutated, pretreated metastatic breast cancer, yielding partial responses in approximately 17% of cases and a median progression-free survival of 3.9 months [215].

In addition to its direct cytotoxicity, trabectedin exhibits strong immunomodulatory properties. When combined with IL-12, it activates NK cells to release chemokines such as CCL5 and XCL1, facilitating the recruitment of CXCL10-producing cDC1 cells and enhancing intratumoral infiltration of CD8^+^ T lymphocytes [216]. This immune remodeling improves tumor responsiveness to anti-PD-L1 therapy and leads to enhanced tumor regression.

### 5.2. Microbial Products: Rapamycin, Actinomycin D, and Salinomycin

Rapamycin, first identified on Easter Island as an antibiotic derived from the aerobic Gram-positive bacterium *Streptomyces hydroscopicus*, exhibits dual biological relevance as both a potent inhibitor of fungal proliferation and an immunosuppressive and anticancer compound in humans [217]. Its anticancer activities are primarily linked to suppression of the mammalian target of Rapamycin signaling pathway, where it significantly reshapes crucial gene–miRNA regulatory networks in TNBC cells [218]. This includes the upregulation of c-MYC, MAPK1, PIK3R1 and miR-92a, miR-16, and miR-20a, together with the downregulation of PTGS2, EGFR, VEGFA and miR-146a and miR-145, thereby reshaping cancer-related, cell cycle, and transcriptional programs. Functionally, these molecular alterations translate into reduced cell migration, diminished wound-healing capacity, and enhanced apoptosis, indicative of suppressed metastatic behavior. Rapamycin alone demonstrates antiproliferative effects and promotes tumor cell death, with these cytotoxic outcomes being further intensified when combined with *Ilex latifolia* [146]. Its therapeutic relevance is further supported by evidence showing that joint administration with EGFR inhibitors reduces cell survival, augments apoptosis, and produces synergistic antitumor responses in TNBC, suggesting that inhibition of mTOR can enhance the responsiveness of TNBC to EGFR-targeted therapy [219]. Moreover, combining rapamycin with cyclophosphamide has been shown to significantly decrease tumor burden and metastatic spread while improving survival outcomes; this enhanced benefit may relate to hypoxic tumor conditions where HIF-1α contributes to rapamycin’s anticancer actions, whereas cyclophosphamide may counteract rapamycin-induced AKT feedback activation, together resulting in superior tumor suppression and metastasis control [220]. Parallel strategies targeting the mTOR pathway through the concurrent use of itraconazole and rapamycin reveal synergistic inhibition of TNBC proliferation and motility, with flow cytometry demonstrating G0/G1 arrest and blockade of G1/S transition [221]. Beyond rapamycin, Actinomycin D—isolated from *Streptomyces parvulus* associated with a marine sponge from Barrang Lompo Island, Makassar, Indonesia—also represents an important therapeutic candidate, as it specifically downregulates Sox-2, leading to depletion of CSC populations and subsequently impairing tumor-initiating capability [222]; in addition, Actinomycin D exhibits synergism with doxorubicin through P53-dependent apoptotic activation, reinforcing its relevance in TNBC treatment [223]. Salinomycin, a naturally occurring polyether ionophore synthesized by *Streptomyces albus* [224], has similarly gained prominence, particularly when co-administered with Budesonide, where it synergistically suppresses TNBC growth by triggering intrinsic apoptosis, increasing intracellular ROS 2–3 times, inducing 25–30% mitochondrial depolarization, enhancing autophagy via AKT/mTOR inhibition, and reducing migration and stemness through EMT modulation [225]. Salinomycin further promotes anoikis sensitivity with associated caspase-3 and caspase-8 activation and Poly (ADP-Ribose) Polymerase cleavage, while concurrently inhibiting migration and invasion through downregulation of MMP-9 and MMP-2 mRNA, and markedly suppresses IL-6-triggered STAT3 activation [226]. Supporting these findings, chemically modified analogs of salinomycin reported by Malekmarzban [226] reduce tumor cell viability, metastasis, and angiogenesis in TNBC, potentially through modulation of the Wnt pathway, although additional studies are required to clarify their effects on CSC, healthy cells, and in vivo performance. Notably, these mechanistic effects translate into a selective inhibitory action of salinomycin on CSC in TNBC, where increasing concentrations correlate with progressive loss of cell viability [227].

### 5.3. Animal-Derived Anticancer Agents

Beyond marine and microbial sources, the animal kingdom has historically provided important bioactive compounds with clinically relevant anticancer properties, and emerging evidence continues to identify novel animal-derived peptides and proteins exhibiting selective activity against TNBC. These molecules often exploit structural features refined through evolution to target cellular membranes, disrupt mitochondrial function, or modulate immune surveillance mechanisms, offering unique therapeutic opportunities.

Among the most extensively studied animal-derived candidates are peptide toxins isolated from venomous organisms. Melittin, the principal component of European honeybee (*Apis mellifera*) venom, has demonstrated significant antiproliferative effects in TNBC models through multiple mechanisms. Mechanistic studies reveal that melittin selectively reduces the viability of TNBC cells by inhibiting the activation of EGFR and modulating downstream oncogenic Akt and MAPK signaling pathways [228]. At the membrane level, melittin induces cell death through pore formation and subsequent caspase 3-mediated apoptosis. Notably, in vivo studies using immune-competent murine models of aggressive TNBC have shown that combination therapy with melittin and docetaxel significantly reduces tumor growth compared to either agent alone, an effect associated with marked reduction in tumor cell proliferation (Ki-67) and enhanced DNA fragmentation (TUNEL assay) [228]. Importantly, melittin treatment—both individually and in combination—reduced the population of cancer cells expressing PD-L1, thereby alleviating immune suppression within the TNBC microenvironment and potentially restoring T-cell-mediated antitumor activity. These findings position melittin as a dual-function agent capable of enhancing chemotherapy efficacy while counteracting immune evasion.

Beyond venom components, host defense peptides such as lactoferricin B—a peptide derived from bovine milk protein lactoferrin—exhibit selective antitumor activity against TNBC. Comprehensive evaluation of lactoferricin B across breast cancer cell lines representing different molecular subtypes demonstrated significant apoptosis induction in TNBC lines MDA-MB-231 and MDA-MB-468, with the peptide showing minimal toxicity toward normal breast epithelial cells (MCF-10A) [229]. Lactoferricin B also inhibited the invasive capacity of TNBC cells in vitro. These findings underscore lactoferricin B as a promising and safe candidate for TNBC therapy, with the translational advantage of being derived from a widely available dietary source.

Animal-derived aminosterols isolated from dogfish shark liver (*Squalus acanthias*) have also been investigated for their anticancer properties. Squalamine, a natural aminosterol compound, disrupts ion transport and inhibits angiogenesis by blocking endothelial cell proliferation and migration [230]. A comprehensive review by Sterling et al. [231] highlights the role of squalamines in blocking tumor-associated angiogenesis and cancer progression, with evidence supporting their capacity to enhance tumor sensitivity to chemotherapy through modulation of the TME. Although initial clinical development of squalamine in breast cancer encountered challenges related to formulation and pharmacokinetics, these aminosterols continue to inform the design of synthetic analogs with improved therapeutic properties.

The growing recognition of animal-derived peptides as modulators of antitumor immunity further supports their therapeutic relevance. The cell-penetrating properties of crotamine, a peptide derived from South American rattlesnake venom, have been exploited for the intracellular delivery of therapeutic molecules in cancer models, with recent investigations exploring its potential in oncologic applications [232]. Peptide engineering approaches have also been applied to melittin itself, where attachment of a cancer-specific RGD ligand enhanced selectivity toward cancer cells by directing binding to integrins overexpressed on TNBC membranes and tumor-associated vasculature. These observations underscore the potential of animal-derived scaffolds not only as direct cytotoxic agents but also as multifunctional platforms for drug delivery and immunotherapy combinations, with ongoing research focused on optimizing selectivity, bioavailability, and safety profiles for clinical translation. 

## 6. Overcoming Drug Resistance with Natural Compounds

### 6.1. Mechanisms of Resistance in TNBC

TNBC is highly prone to developing chemoresistance, primarily due to increased drug efflux mediated by ABC transporters [26]. Among these, P-glycoprotein is a transmembrane glycoprotein that actively pumps a wide range of chemotherapeutic agents out of cancer cells, and its overexpression is strongly correlated with poor prognosis, reduced patient survival, and multidrug resistance (MDR) in TNBC. Both clinical and experimental evidence indicate that chemotherapy can induce Pgp expression, and targeting Pgp through inhibitors, lysosomal modulation, or nanoparticle-based strategies can help restore sensitivity to anticancer drugs [233]. In addition, ABCC1, ABCG2, and ABCC11 are markedly upregulated in TNBC relative to other breast cancer subtypes, and their expression is further enhanced by activation of the hedgehog signaling pathway [234]. These transporters exhibit broad and overlapping substrate specificity: ABCC1 mediates resistance to anthracyclines, taxanes, mitoxantrone, and methotrexate; ABCG2 exports drugs including 5-fluorouracil, methotrexate, doxorubicin, irinotecan, and mitoxantrone; and ABCC11 confers resistance to 5-fluorouracil and methotrexate, collectively compromising the effectiveness of conventional TNBC chemotherapy [234]. ABCG2 is critical for chemoresistance in TNBC stem-like cells, and its inhibition via growth hormone receptor blockade sensitizes cells to treatment [235]. Although strategies targeting ABC transporters through activity or expression inhibition exist, early-generation inhibitors were limited by toxicity, poor selectivity, and inadequate effects on intracellular drug accumulation [236]. Beyond transporter-mediated drug efflux, chemoresistance in TNBC is reinforced by phenotypic reprogramming processes, particularly EMT and the emergence of CSC populations. EMT facilitates a mesenchymal phenotype, enhancing metastatic potential, promoting survival in the heterogeneous TME, reducing therapeutic efficacy, and generating CSC populations [38]. TGF-β signaling plays a central role in regulating EMT, stemness, and apoptosis, with overexpression of TGF-β1 and TGF-βR1 observed in CSC-enriched subpopulations of TNBC cells [237]. Following chemotherapy, these EMT-associated stem-like subpopulations frequently survive treatment, driving tumor recurrence and sustained chemoresistance. Moreover, chemotherapy-induced hypoxia stabilizes HIFs, which further amplify CSC enrichment through IL-6 and IL-8 signaling, contributing to acquired chemoresistance [237]. Overall, TNBC chemoresistance arises from the combined effects of ABC transporter upregulation, EMT induction, CSC maintenance, and hypoxia-driven signaling, underscoring the need for integrated therapeutic strategies that combine selective transporter inhibition with advanced drug delivery systems such as nanoparticle- or nanodiamond-conjugated therapeutics.

### 6.2. Natural Product Strategies

#### 6.2.1. Inhibiting ABCG2/MDR1 Pumps

A predominant strategy by which natural compounds are able to overcome drug resistance in cancer is through the inhibition of key efflux pumps, particularly ABCG2 and MDR1/P-gp, thereby enhancing intracellular drug accumulation and restoring chemotherapeutic sensitivity. In this context, the sesquiterpene lactone helenalin and its derivatives bis(helenalinyl)malonate (BHM) and bis(helenalinyl)glutarate (BHG) exhibit remarkable inhibitory effects against TNBC, where they significantly suppress the activity of ABCB1 and ABCG2 transporters in resistant cell models [238]. In addition to efflux pump blockade, helenalin predominantly induces G2/M-phase arrest, while BHM and BHG robustly trigger apoptosis, reflected by increased ROS production, elevated caspase-9 activity, accumulation of sub-G1 DNA, and specific inhibition of DNA topoisomerase II in the case of BHG. These combined actions result in tumor growth inhibition and potential prevention of MDR progression, with cytotoxic effects influenced by target expression levels. Supporting these findings, alantolactone further contributes to resistance modulation by suppressing STAT3 and NF-κB nuclear signaling in MDA-MB-231 cells [238]. Beyond sesquiterpene lactones, numerous natural compounds—particularly flavonoids—demonstrate strong promise as MDR1/ABCG2 inhibitors because of their selectivity and low toxicity, showing comparable efficacy to classical inhibitors [239]. Hesperetin effectively reverses P-gp-mediated resistance, kaempferol downregulates ABCB1 and ABCC1 expression, and curcumin inhibits ABCG2-dependent MDR in vitro. Additional natural agents, including BBR, licochalcone, lignans, honokiol, and quercetin, also interfere with ABC transporter function or expression [240]. Similarly, tetrandrine increases intracellular chemotherapeutic retention by modulating MDR1/P-gp expression, whereas oxymatrine and matrine reverse resistance through apoptosis induction, MRP1 suppression, and NF-κB inactivation [240].

#### 6.2.2. Targeting CSC Markers

CD44, a type I transmembrane glycoprotein, is widely recognized as a hallmark of CSCs, and its elevated expression is closely associated with therapeutic failure in breast cancer due to its involvement in resistance-driven signaling cascades [241]. Numerous natural bioactive molecules interfere with CD44 activity and disrupt CSC maintenance pathways. Quercetin markedly reduces the CD44^+^/CD24^−^ stem-like breast cancer subset, thereby limiting tumor initiation, progression, and resistance to chemotherapy, while resveratrol and EGCG similarly suppress CD44 expression and attenuate CSC characteristics [242]. Curcumin achieves CD44 repression through inhibition of STAT3 phosphorylation, diminished STAT3 DNA-binding ability, and weakening of NFκB–STAT3 interactions, whereas isoharringtonine likewise decreases the proportion of CD44^+^ cells [183]. Concurrently, aldehyde dehydrogenases (ALDHs), which play crucial roles in detoxification and oxidative stress defense, contribute to CSC persistence and therapeutic resistance by oxidizing aldophosphamide and neutralizing chemotherapeutic agents [241]. Natural compounds also act effectively on this pathway: curcumin also reduces ALDH1A1 expression along with CD44 in TNBC-derived CSCs, while agents such as machilin D, celastrol, and triptolide decrease CD44^+^/CD24^−^ and ALDH1^+^ cell populations, suppress mammosphere formation, and downregulate ALDH1 levels [183]. Additional molecules, including psoralidin, which functions through γ-secretase inhibition, caffeic acid, which lowers CD44 and ALDH1 expression despite limited cytotoxicity, and *Ganoderma lucidum* extracts, which reduce ALDH1 and CD44^+^ CSCs, further highlight the therapeutic significance of natural products in simultaneously targeting CD44 and ALDH1 to mitigate breast cancer stemness and treatment resistance [243].

#### 6.2.3. Modulation of Redox Balance

Modulating cellular redox homeostasis has gained prominence as a promising approach to counteract therapeutic resistance in several cancer types [244]. Redox equilibrium relies on the precise control of ROS generation—primarily arising from the mitochondrial electron transport chain, endoplasmic reticulum oxidases, and NADPH oxidases—together with efficient ROS detoxification by endogenous antioxidant defenses, including enzymatic and nonenzymatic antioxidants, redox-regulatory proteins, and transcriptional factors such as Nuclear factor erythroid 2 (NRF2) [245]. NRF2 is particularly crucial, as it coordinates antioxidant responses while also regulating drug metabolism, cellular energetics, and proteasomal degradation [246]. Within this framework, numerous bioactive compounds exert therapeutic effects through differential modulation of ROS signaling [247].

Resveratrol exhibits robust antioxidant activity by scavenging ROS, activating the Nrf2/HO-1 axis, reducing lipid peroxidation, increasing superoxide dismutase (SOD) activity even at low doses, and upregulating SOD2 expression in cancer cells [248]. In contrast, other natural agents demonstrate anticancer potential through ROS induction and oxidative stress-mediated cytotoxicity. For example, diosgenin suppresses TNBC stem cell growth by activating caspase 3/7–dependent apoptosis alongside ROS elevation [243]. Similarly, hydroxycinnamic acid derivatives from *Cynara scolymus* reduce cell proliferation through increased ROS and stimulation of the ROS/Nrf2 pathway [246]. Acidic *Pleurotus abalonus* polysaccharide also elevates ROS and promotes apoptotic cell death, while butein derived from *Rhus verniciflua* and epicatechin from *Theobroma cacao* enhance ROS production and thereby inhibit cancer cell proliferation [246]. Modulation of antioxidant defenses or deliberate enhancement of oxidative stress thus represents complementary redox-centered mechanisms capable of shaping cancer cell survival and therapeutic responsiveness.

#### 6.2.4. Re-Sensitizing Cells to Taxanes and Anthracyclines

Chemoresistance to frontline agents such as anthracyclines and taxanes represents a major therapeutic limitation in TNBC, a process strongly associated with the expansion of tumor cell subpopulations exhibiting stem-like characteristics [249]. Although TNBC can initially respond to these chemotherapies, complete remission is achieved in fewer than 30% of cases, and the disease is still marked by elevated recurrence and mortality rates [200]. Consequently, there is growing interest in adjunct strategies capable of overcoming resistance, including the use of natural compounds such as β-lapachone combined with hydroxytyrosol, which has demonstrated notable anticancer activity against TNBC cells and resistant variants [249]. As previously discussed, numerous natural bioactives enhance the efficacy of conventional chemotherapeutics by acting synergistically and restoring drug sensitivity [250].

#### 6.2.5. Integrative Synthesis and Prioritization of Therapeutic Strategies

The accumulated preclinical and translational evidence highlights that despite the diversity of natural compounds investigated in TNBC, a limited subset emerges as particularly promising based on mechanistic convergence and translational relevance. Polyphenols such as curcumin, resveratrol, and EGCG consistently demonstrate multitarget activity across interconnected pathways governing tumor metabolism, inflammatory signaling, EMT, and cancer stem cell maintenance, thereby addressing key drivers of TNBC aggressiveness and therapeutic resistance. Similarly, agents targeting central signaling hubs—including mTOR (rapamycin), microtubule dynamics (paclitaxel), and DNA repair vulnerabilities (trabectedin)—appear especially actionable, given their capacity to synergize with standard chemotherapeutics and immunomodulatory strategies. From a mechanistic standpoint, pathways regulating CSC plasticity, EMT-associated drug tolerance, ABC transporter-mediated efflux, and redox homeostasis emerge as critical therapeutic nodes, as they directly underlie chemoresistance and disease recurrence. Importantly, compounds exerting pleiotropic effects on these mechanisms, particularly when deployed in combination or delivery-enhanced approaches, are more likely to overcome the limitations associated with single-target interventions. In contrast, natural products with narrow specificity or pronounced pharmacokinetic constraints may face greater barriers to clinical translation despite strong preclinical efficacy, underscoring the need for prioritization based on mechanistic breadth, combinatorial potential, and translational feasibility.

## 7. Immunomodulatory Potential of Natural Compounds

### 7.1. Modulation of PD-1/PD-L1 Axis

Factors within the TME, together with suppressive cytokines, promote T-cell exhaustion, and PD-L1 constitutes a principal contributor to this immunosuppressive state [250]. PD-1 and PD-L1 are immunoglobulin superfamily transmembrane proteins whose interaction dampens antitumor immunity, thereby sustaining immune escape and contributing to the suboptimal performance of various immunotherapies [250]. Consequently, increasing attention has focused on natural products capable of targeting this checkpoint, and several herbal compounds have demonstrated inhibitory activity against PD-1/PD-L1 binding. Active constituents from *Geranii Herba* and quercetin effectively suppress this interaction, while *Toxicodendron vernicifluum* (*Rhus verniciflua* Stokes), rich in flavonoids and polyphenols, produces a strong, dose-dependent blockade [251]. Caffeoylquinic acids, particularly mono-CQAs, partially disrupt the checkpoint system, and ellagic acid derived from *Rubus coreanus* also exhibits dose-dependent inhibition [251]. Further promising evidence derives from Glyasperin C isolated from *Glycyrrhiza uralensis*, which produces approximately 30–65% inhibition of this interaction at 100 μM, and *Salvia plebeia* extract, which has been shown to prevent PD-1/PD-L1 binding in ELISA-based analyses [252,253].

### 7.2. Enhancement of Dendritic Cell and T-Cell Function

Dendritic and T-cell function plays an essential role in orchestrating antitumor immune responses, as DCs coordinate antigen uptake, maturation, and T-cell activation, while T cells mediate cytotoxic and regulatory immune activities. Increasing evidence demonstrates that polysaccharides—classified into fungal, plant, lichen, algal, animal, and bacterial types—can bind to pattern recognition receptors on DCs, activating PI3K/AKT, MAPK, NF-κB, Dectin-1/Syk and related signaling pathways to enhance DC maturation, metabolism, antigen presentation, and T-cell stimulation, ultimately exerting antitumor effects [254]. However, T-regulatory cells, a specialized T-cell subset, suppress antitumor immunity by restraining effector T-cell proliferation and cytokine release [255]. Several natural compounds have emerged as promising immunomodulators capable of counteracting tumor-driven immune suppression and strengthening adaptive immunity. Resveratrol enhances CD4+ and CD8+ T-cell proliferation and activation, boosting their antitumor capacity [27], while curcumin modulates key immune components including DCs, macrophages, B and T lymphocytes, cytokines, and transcription factors, influencing downstream signaling to support immune regulation [256,257]. Pterostilbene stimulates IL-1β and IL-18 secretion, recruits immune cells targeting CSCs, strengthens antitumor CD44+ and CD8+ T cells, NK cells and B cells, reduces tumor-supportive Tregs, and M2-TAMs, promotes macrophage polarization toward an M1 phenotype, and enhances Th1-type immune responses, thereby destabilizing the CSC protective niche [242]. Similarly, Wogonin, a *Scutellaria ocmulgee* leaf extract, inhibits TGFβ1 secretion, disrupting TGFβ1-mediated Treg enhancement and reversing tumor-induced immunosuppression, potentially restoring immune capacity to eliminate CSCs. Baicalin and Baicalein further contribute by promoting M1-like TAM polarization, suppressing M2-TAMs, enhancing T-cell responses, and regulating the Treg/Th17 balance to counter CSC-associated immune evasion [205]. Finally, Tussilagone, from *Tussilago farfara plant,* has been shown to enhance CD8+ T-cell activation, reinforcing cytotoxic immune function against tumors [258].

### 7.3. Opportunities for Combination with Immune Checkpoint Inhibitors

Natural compounds with immunomodulatory and/or cytotoxic properties represent promising candidates for combination with immune checkpoint inhibitors (ICIs) in TNBC, particularly in early-stage disease, where tumors appear to be more immunologically active than metastatic counterparts. Clinical experience in TNBC indicates that ICI monotherapy has shown limited efficacy in advanced or metastatic settings, whereas early-stage, treatment-naïve tumors can exhibit meaningful immune activation, including increased tumor-infiltrating lymphocytes and upregulation of PD-L1 following short-term ICI exposure [259].

These observations support the concept of immune priming prior to cytotoxic therapy, suggesting that brief ICI-based interventions may sensitize tumors to subsequent treatments. In this context, natural compounds capable of enhancing antitumor immunity, reducing immunosuppression, or modulating inflammatory signaling may act as synergistic partners, amplifying the depth and durability of immune responses [260]. Such combinations are conceptually aligned with existing therapeutic paradigms, as demonstrated by the clinical success of ICIs combined with poly(ADP-ribose) polymerase inhibitors, which exert immunomodulatory effects by reshaping the tumor immune microenvironment [261].

Translating these combination strategies into clinical practice will require careful patient selection guided by robust predictive biomarkers, alongside comprehensive toxicity assessment to ensure safety and tolerability. Importantly, these approaches should be evaluated within de-escalation frameworks, with the goal of reducing cumulative chemotherapy exposure while preserving—or potentially enhancing—oncologic efficacy. If appropriately validated, combinations of ICIs with selected natural compounds may represent a rational and innovative strategy to optimize immunotherapy outcomes in TNBC.

## 8. Challenges and Perspectives for Clinical Translation

### 8.1. Bioavailability, Pharmacokinetics, and Toxicity

To date, the majority of evidence supporting the therapeutic potential of natural compounds and combination strategies in TNBC is derived from in vitro and in vivo preclinical models, while robust clinical validation remains limited. Consequently, efforts toward clinical translation are frequently hindered by pharmacological and pharmacokinetic constraints. Many of these molecules exhibit low water solubility and poor membrane permeability, restricting systemic bioavailability. Inefficient gastrointestinal absorption further reduces the fraction of active compound reaching circulation, particularly for large, hydrophobic molecules such as polyphenols, alkaloids, and flavonoids [262]. Rational structural modification represents one strategy to partially overcome these limitations. For example, chrysin, a flavonoid with broad antitumor activity, is hindered by poor aqueous solubility and low systemic bioavailability; however, incorporation of the 1H-benzimidazole-4-carboxamide pharmacophore from the orally bioavailable PARP1 inhibitor veliparib into the chrysin scaffold generated derivatives with enhanced biological activity, including antiproliferative effects in BRCA-deficient breast cancer cells and tumor growth inhibition following oral administration in xenograft models, supporting the translational potential of bioavailability-oriented optimization [262]. Similarly, resveratrol demonstrates poor solubility, limited absorption, and rapid hepatic metabolism, which further constrain its therapeutic potential [146]. Similarly, curcumin and EGCG are characterized by low bioavailability and fast metabolic clearance, with EGCG additionally exhibiting stability issues that contribute to discrepancies between in vitro and in vivo outcomes [26,263]. PTX suffers from poor solubility and dose-dependent immunotoxicity, hypersensitivity, and neurotoxicity, while CPT is constrained by unfavorable physicochemical properties, rapid clearance, unpredictable distribution, and hematologic and gastrointestinal toxicities [264].

Inter-individual variability further complicates pharmacokinetic predictions. Genetic polymorphisms, diet, gut microbiota composition, and concomitant medications can influence absorption, distribution, metabolism, and excretion, particularly in patients with comorbidities or polypharmacy. Conventional pharmacokinetic protocols, optimized for synthetic drugs, are often insufficient for complex natural compounds [262]. Notably, emerging evidence underscores the role of the gut microbiota as a major contributor to this variability, modulating bioavailability, as microbial metabolism of polyphenols and flavonoids can generate bioactive metabolites, affecting systemic exposure and therapeutic efficacy [262]. Safety considerations also require careful evaluation prior to clinical use. Some natural products have been associated with hepatotoxicity and mild gastrointestinal adverse effects, highlighting the necessity of phase I dose escalation studies to establish maximum tolerated doses and inform subsequent phase II trials [241].

Importantly, while preclinical studies provide essential mechanistic insights and proof-of-concept evidence, their translation into clinical benefit remains inherently complex. Experimental models do not fully recapitulate the heterogeneity of human TNBC, nor the influence of the TME, immune system interactions, and host-related factors that shape therapeutic responses in patients. In addition, differences in metabolism, systemic exposure, and toxicity profiles between animal models and humans may lead to discrepancies between preclinical efficacy and clinical outcomes [261]. Furthermore, the clinical literature is characterized by a predominance of positive preclinical reports, while negative or neutral clinical outcomes remain underreported. Consistent with these limitations, several natural compounds that demonstrated robust anticancer activity in vitro failed to achieve meaningful clinical efficacy due to subtherapeutic systemic exposure, dose-limiting toxicity at pharmacologically relevant concentrations, or lack of target engagement in humans. These inconsistencies underscore the limitations of reductionist experimental systems and highlight the need for transparent reporting of negative findings, realistic dosing strategies, and early integration of pharmacokinetic and pharmacodynamic endpoints in clinical trial design. Consequently, promising preclinical findings should be interpreted with caution and validated through rigorously designed clinical studies integrating pharmacokinetic, safety, and biomarker-driven endpoints. Accompanying intrinsic toxicity, natural compounds may exert clinically relevant drug–drug interactions through modulation of cytochrome P450 enzymes, drug transporters, and immune signaling pathways. Such interactions are particularly relevant in TNBC patients receiving multi-agent chemotherapy, corticosteroids, or immunotherapies, where unintended pharmacokinetic or immunological interference may compromise efficacy or safety [261]. These risks reinforce the necessity of systematic interaction studies and controlled combination trials, rather than empirical supplementation alongside standard treatments.

### 8.2. Nanocarrier-Based Delivery Systems

Since the clinical application of natural compounds in cancer therapy is often hindered by intrinsic limitations, novel approaches involving the encapsulation of bioactive molecules within NPs or microparticles have been developed. Nanotechnology-driven drug delivery systems take advantage of the distinct properties of materials at the nanoscale, including their small size, high drug-loading capacity, precise delivery potential, and structural stability. These features collectively enhance the therapeutic efficacy of loaded drugs [265]. Such systems offer several benefits in oncology, including controlled and sustained drug release, selective tumor targeting, and extended systemic circulation, as their hydrophobic nature reduces clearance by the reticuloendothelial system [266]. Nanodrug delivery systems (NDDSs) employ a variety of strategies, such as passive and active targeting, stimuli-responsive release, co-delivery of multiple agents, and multimodal approaches, thereby broadening their therapeutic versatility [196].

Building on these properties, recent research has highlighted the ability of NDDSs to further enhance the solubility, stability, and overall therapeutic performance of chemotherapeutic drugs. For example, encapsulating EGCG in NPs has been shown to improve both its stability and bioavailability, effectively addressing its key pharmacokinetic limitations [263]. Xiong et al. [266] engineered PTX–curcumin NPs using a poly(ε-caprolactone)-poly(ethylene glycol)-poly(ε-caprolactone) nanocarrier, enabling the co-delivery of PTX and curcumin. This approach improved drug solubility and significantly inhibited tumor progression compared with controls. Similarly, chondroitin sulfate-conjugated poly(lactic-co-glycolic acid) lipid–polymer hybrid nanoparticles targeting CD44 on TNBC cells increased cellular uptake and induced marked cytotoxic effects. These NPs promoted apoptosis by modulating the Wnt/β-catenin pathway and apoptotic proteins [267].

Targeting CSCs is another innovative strategy. Nanoparticles coated with hyaluronic acid, a ligand for CD44, have demonstrated selective delivery of chemotherapeutics to breast CSCs while sparing normal cells, with efficacy confirmed both in vitro and in vivo [234]. Xu et al. [264] advanced this concept by designing ROS-responsive NPs that co-assemble a ROS-sensitive prodrug (CTF) with hyaluronic acid-modified IR780, a near-infrared photosensitizer. These “nano-bomb” NPs integrate chemotherapy with photodynamic and photothermal therapy, inducing immunogenic cell death, enhancing antitumor immune responses, eradicating primary tumors, and suppressing metastasis through cycles of ROS generation and tumor cell elimination.

Beyond tumor- and cell-specific delivery, nanocarriers have been engineered to overcome the BBB and target brain metastases, a major challenge in breast cancer therapy. Approaches include ligand-decorated nanoparticles that exploit receptor-mediated transcytosis (e.g., transferrin, insulin, or LDL receptors) to cross the BBB, as well as hybrid or biomimetic systems that combine BBB transport with selective tumor targeting. These strategies enhance drug accumulation in metastatic brain lesions, improve therapeutic efficacy, and reduce off-target toxicity, highlighting the potential of nanomedicine for treating brain metastases in preclinical models [110].

Resveratrol-based nanotherapeutics further illustrate the theranostic potential of NDDSs. Nanoformulations of resveratrol enable tumor-targeted imaging alongside treatment, providing improved image-guided therapy for TNBC [268]. NDDSs have also been developed to enhance the bioavailability of resveratrol, addressing its poor water solubility and pharmacokinetic limitations [146]. Interestingly, combining resveratrol with short, high-intensity electrical pulses in TNBC cells has been shown to increase cellular uptake, elevate ROS production, and induce robust apoptosis, demonstrating the therapeutic potential of integrated strategies to overcome bioavailability issues [269].

Despite their promising advantages, nanotherapeutics face challenges. Nanomedicines based on inorganic materials may inadvertently promote metastasis by opening endothelial gaps that facilitate cancer cell intravasation. Additionally, lysosomal degradation can reduce the stability and efficacy of nanotherapeutic agents, emphasizing the importance of careful design and optimization to maximize their therapeutic benefits [265].

### 8.3. Standardization of Extracts: Regulatory Hurdles

The clinical translation of natural compounds for TNBC is hindered by challenges in extract standardization and regulatory compliance. Natural extracts exhibit considerable variability in bioactive content due to differences in plant source, harvesting conditions, extraction methods, and storage, which complicates reproducibility and therapeutic consistency across studies and clinical batches. Such variability undermines efforts to define dose–response relationships and to ensure comparable pharmacokinetic and pharmacodynamic profiles, both of which are critical for regulatory approval [270].

Moreover, regulatory agencies such as the U.S. Food and Drug Administration and European Medicines Agency require validated quality control methods and clear identification of active constituents—a requirement that is inherently difficult for complex multi-component botanical extracts compared with single-molecule drugs [271]. The absence of standardized compositions and well-defined molecular signatures further complicates clinical trial design, limiting comparability between studies and delaying the generation of high-quality safety and efficacy data necessary for product registration.

Addressing these challenges will require the integration of advanced analytical platforms—such as metabolomics, chemometric fingerprinting, and quantitative marker-based standardization—together with the development of fixed-composition extracts and harmonized regulatory frameworks, thereby facilitating reproducibility, regulatory acceptance, and the clinical translation of natural compound-based therapies for TNBC [272].

### 8.4. Clinical Trial Landscape and Future Prospects

Although preclinical research demonstrates the antitumor potential of natural compounds in TNBC in vitro and in animal models, clinical investigations remain limited and largely exploratory [273]. Natural compounds in TNBC engage diverse molecular mechanisms, yet only a subset demonstrates meaningful translational potential. Pathways influencing resistance reversal, immune modulation, and TME interactions are particularly promising, whereas isolated in vitro effects often lack clinical relevance. Effective translation requires prioritizing mechanistic depth, dose-appropriate strategies, and integration with clinically meaningful endpoints over exhaustive mapping of signaling pathways. Emerging evidence frames these agents not as universal anticancer drugs, but as context-dependent modulators whose efficacy hinges on tumor molecular state, immune landscape, and resistance phenotype. Many clinical failures stem from absent biomarker-guided stratification, inappropriate preclinical dosing, and oversimplified assumptions of target engagement. Rational development should therefore align compound selection with defined resistance mechanisms—such as ABC transporter overexpression, redox imbalance, or cancer stem cell enrichment—and specific immune profiles, rather than applying natural agents indiscriminately across heterogeneous TNBC populations.

As discussed above, translation to clinical benefit has been hindered by chemical diversity, incomplete understanding of pharmacokinetics and pharmacodynamics, and variability in natural product composition due to cultivation, seasonality, and harvesting conditions [272]. Successful clinical translation further depends on rigorous quality control, reproducibility, and predictive modeling, as promising preclinical results do not always correspond to clinical outcomes [274]. Collaborative efforts across research, clinical, and regulatory sectors are essential to ensure mechanistic understanding, standardized methodologies, and integrated approaches throughout the translational pipeline [275].

Building on the delivery strategies discussed above, innovative formulations should be developed to address the aforementioned challenges. Nanotechnology-based systems—such as nanoencapsulation, nanomicelles, nanosizing, and liposomes—enhance solubility, stability, and bioavailability while allowing for selective tumor targeting. Structural modifications and prodrug approaches further optimize pharmacokinetics, and self-assembled or natural material-based nanoparticles improve biocompatibility and biodegradability compared with conventional nanomaterials [274].

Mechanistic insights have advanced through integrated omics techniques (genomics, transcriptomics, proteomics, metabolomics, and metagenomics), providing a comprehensive view of molecular actions. Structural biology methods, including X-ray crystallography, molecular docking, and site-directed mutagenesis, elucidate binding mechanisms, while AI, machine learning, and systems biology facilitate bioactivity prediction, experimental optimization, and development of personalized therapies [274]. Improved preclinical models, such as 3D cultures, organoids, humanized mice, and patient-derived xenografts, better recapitulate human physiology, enhancing predictive relevance for clinical outcomes.

Emerging therapeutic strategies increasingly emphasize precision medicine, leveraging tumor heterogeneity to maximize efficacy and minimize side effects. Natural products combined with immunotherapy can modulate immune responses to enhance antitumor activity, and integration with conventional chemotherapeutics or targeted agents may improve efficacy while reducing toxicity [247]. Ongoing clinical research is elucidating regulatory pathways and potential incorporation of phytochemicals into standard regimens, offering less invasive and more tailored therapeutic options.

Within this precision-medicine framework, TNBC-specific trials remain scarce. Persistent variability in formulations, low bioavailability, and the lack of standardized dosing highlight the need for well-designed Phase II and III trials assessing both clinical outcomes and mechanistic biomarkers [273]. Combined with advances in nanotechnology, prodrug development, mechanistic characterization, and preclinical modeling, such studies are crucial to establish precise, effective, and individualized therapeutic options. Improved understanding of natural products may ultimately allow for better prediction of clinical outcomes and enhanced quality of life for patients with TNBC [247].

## 9. Conclusions

TNBC represents one of the most aggressive and therapeutically challenging subtypes of breast cancer, defined by marked molecular heterogeneity, rapid disease progression, and the absence of effective targeted therapies. Conventional treatment strategies, including chemotherapy and selected targeted agents, are frequently limited by intrinsic or acquired resistance, cumulative toxicity, and high rates of recurrence. Within this complex therapeutic landscape, natural products have emerged as a compelling strategy due to their capacity to simultaneously modulate multiple oncogenic hallmarks of TNBC.

Bioactive compounds spanning polyphenols, terpenoids, alkaloids, and marine-derived molecules exert pleiotropic effects that interfere with sustained proliferative signaling, apoptotic evasion, angiogenesis, immune suppression, invasion and metastasis, and metabolic rewiring. This multitarget activity is particularly advantageous in TNBC, where single-pathway inhibition often fails to achieve durable clinical responses. Rather than acting as isolated cytotoxic agents, natural compounds function as network modulators capable of reshaping dysregulated signaling ecosystems.

An important insight emerging from recent studies is the ability of natural compounds to overcome key mechanisms of therapeutic resistance. Through modulation of ATP-binding cassette transporters, targeting of cancer stem-like cell populations, and regulation of redox homeostasis, these agents can restore sensitivity to conventional chemotherapies. Their immunomodulatory properties—including regulation of PD-1/PD-L1 signaling and enhancement of T-cell and dendritic cell function—further support their use in combination with immune checkpoint inhibitors, reinforcing their value within integrated chemo-immunotherapeutic strategies. Without rigorous clinical validation, standardized formulations, and biomarker-driven deployment, continued accumulation of preclinical evidence alone is unlikely to translate into clinical impact.

In summary, natural products represent a versatile yet underutilized resource in TNBC therapy, with the capacity to target multiple dysregulated pathways, counteract resistance mechanisms, and complement existing oncological treatments. Their rational integration into precision oncology—supported by molecular characterization and advanced delivery technologies—holds promise for the development of more effective, less toxic, and individualized therapeutic regimens. Bridging the rich legacy of natural product pharmacology with modern molecular oncology may ultimately expand the therapeutic arsenal against TNBC, offering new opportunities to improve clinical outcomes and long-term patient survival.

## Figures and Tables

**Table 1:** Key biomarkers associated with TNBC hallmarks and biological features.

TNBC Hallmark	Key Biomarkers	Clinical Outcome
High proliferation	Ki-67 (MKI67) [51]	High tumor growth rate
Basal-like phenotype	CK5/6, CK14, EGFR [52]	Basal epithelial lineage typical of TNBC
DNA repair deficiency	BRCA1, BRCA2, RAD51, PALB2 [53]	Sensitivity to PARP inhibitors and platinum therapy
Genomic instability	TP53 mutations [54]	Very common driver mutation in TNBC
Immune activation	PD-L1 (CD274), CD8, TILs markers (CD3, CD4) [50]	Predicts response to immunotherapy
PI3K/AKT pathway activation	PIK3CA, PTEN loss, AKT1 mutations [55]	Oncogenic signaling pathway
Epithelial–mesenchymal transition	Vimentin, N-cadherin, Snail, Twist [56]	Tumor invasion and metastasis
Angiogenesis	VEGF-A, HIF-1α [57]	Tumor vascularization
Cancer stemness	CD44^+^/CD24^−^ phenotype, ALDH1A1 [58]	Tumor-initiating capacity
Androgen receptor signaling	AR [59]	Luminal androgen receptor subtype

Abbreviations: Ki-67 (MKI67): Marker of proliferation Ki-67; CK5/6: Cytokeratin 5/6; CK14: Cytokeratin 14; EGFR: Epidermal growth factor receptor; BRCA1: Breast cancer susceptibility gene 1; BRCA2: Breast cancer susceptibility gene 2; RAD51: RAD51 recombinase; PALB2: Partner and localizer of BRCA2; TP53: Tumor protein p53; PD-L1 (CD274): Programmed death-ligand 1; CD8: Cluster of differentiation 8; TILs: Tumor-infiltrating lymphocytes; CD3: Cluster of differentiation 3; CD4: Cluster of differentiation 4; PIK3CA: Phosphatidylinositol-4,5-bisphosphate 3-kinase catalytic subunit alpha; PTEN: Phosphatase and tensin homolog; AKT1: AKT serine/threonine kinase 1; VEGF-A: Vascular endothelial growth factor A; HIF-1α: Hypoxia-inducible factor 1-alpha; CD44: Cluster of differentiation 44; CD24: Cluster of differentiation 24; ALDH1A1: Aldehyde dehydrogenase 1 family member A1; AR: Androgen receptor.

**Table 2:** Key diagnostic approaches in triple-negative breast cancer.

Diagnostic Approach	Sensitivity	Specificity	Clinical Application
Mammography [127]	Suboptimal for TNBC	Not specified	TNBC may lack suspicious calcifications; often appears as irregular, ill-defined, spiculated, or round mass; less effective in premenopausal patients with dense breasts; rapid progression may bypass in situ stage
Ultrasound [127]	92–100%	Not specified	High sensitivity; can demonstrate benign-appearing features (round/oval shape, parallel orientation) that may delay diagnosis; features include hypoechogenicity, irregular shape, non-circumscribed margins
Automated Breast Volume Scanning [128]	Superior to ultrasound and mammography	Superior to ultrasound and mammography	Less operator-dependent than hand-held ultrasound; higher reproducibility; provides high-resolution coronal plane imaging; shorter acquisition time
Contrast-Enhanced MRI [127]	~100% for TNBC detection; 85% for DCIS in high-risk women	Not specified for TNBC	Most accurate and sensitive modality for TNBC; features include intratumoral T2 hyperintensity, smooth mass margins, rim enhancement (most accurate predictor of ER status), persistent enhancement pattern; optimal for assessing neoadjuvant chemotherapy response
MRI + Mammography Combined [129]	99% for DCIS in high-risk women	Not specified	Highest sensitivity when combined; no significant gain over MRI alone in women < 40 years or BRCA1 mutation carriers
ctDNA MRD [130]	High sensitivity for recurrence detection	Exceptional specificity	Detects molecular residual disease months before imaging; 95% accuracy for predicting recurrence; enables real-time monitoring of chemotherapy efficacy; identifies very-low-risk groups who may avoid additional chemotherapy
Digital PCR-based ctDNA [131]	High sensitivity	High specificity	Validated in TRICIA trial for risk stratification in residual TNBC; correlates with patient outcomes during capecitabine treatment
Targeted Panel Sequencing (ctDNA) [130]	Lower than MRD assays	High	Less sensitive than tumor-informed MRD assays; may miss detectable ctDNA even when MRD assays are positive
Ki-67 Proliferation Index [132]	Not applicable (predictive)	Not applicable (prognostic)	High expression in TNBC; associated with aggressive disease; helps guide chemotherapy decisions
PD-L1 Expression [132]	Not applicable (predictive)	Not applicable (predictive)	Predicts response to immune checkpoint inhibitors (pembrolizumab, atezolizumab); guides immunotherapy combinations

Abbreviations: ctDNA: circulating tumor DNA; DCIS: ductal carcinoma in situ; ER: estrogen receptor; MRD: molecular residual disease; MRI: magnetic resonance imaging; PCR: polymerase chain reaction; PD-L1: programmed death-ligand 1; TNBC: triple-negative breast cancer.

**Table 3:** Synergistic interactions between natural compounds and conventional chemotherapeutic agents.

Natural Compound	Chemotherapeutic Agent	Main Synergistic Mechanisms	Type of Study
Curcumin	Docetaxel	Reduction in VEGF levels and prevention of docetaxel-associated hematological toxicity	Clinical evidence [152]
	Paclitaxel	Inhibition of NF-κB signaling; induction of programmed cell death; ↓ p53 expression; ↓ VEGF, MMP-9, and ICAM-1	In vitro [150,153]
	Doxorubicin	Enhanced apoptosis; blockade of doxorubicin-induced EMT via PI3K/AKT and TGF-β modulation; S-phase cell cycle arrest	In vitro + In silico [154] In vitro [155]
	Carboplatin	↓ proliferation, invasion, and migration (↓ MMP2/MMP9); impaired DNA repair (↓ RAD51, ↑ γH2AX); ↑ ROS-mediated apoptosis suppression	In vitro [156]
	5-Fluorouracil	Increased susceptibility to apoptosis via NF-κB	In vitro [157]
Resveratrol	Doxorubicin	Enhanced apoptosis and senescence; ↑ intracellular DOX accumulation via downregulation of ABC transporters (MDR1, MRP1); dose-reduction potential	In vitro + In silico [158] In vivo + In vitro [159,160]
	Doxorubicin (oxyresveratrol)	Mitochondrial dysfunction (↓ membrane potential); ↑ ROS generation; DNA damage with cell cycle arrest; activation of caspases-3, -7, -8, and -9	In vitro + In silico [161]
	Salinomycin	Suppression of EMT markers (fibronectin, vimentin, N-cadherin, Slug); inhibition of inflammatory, autophagic, and apoptotic regulators	In vivo + In vitro [162]
	Cisplatin	↓ viability, migration, and invasion via inhibition of TGF-β1–driven EMT; attenuation of PI3K/AKT, Smad, MAPK, and NF-κB signaling; reduced toxicity	In vivo + In vitro [163]
	Paclitaxel	Restoration of paclitaxel sensitivity; induction of senescence and apoptosis, enabling dose reduction	In vitro [164]
	Rapamycin	Induction of apoptosis via mTOR inhibition and prevention of AKT activation	In vitro [165]
Epigallocatechin-3-gallate	Doxorubicin	Prevention of doxorubicin-induced cardiotoxicity	In vivo + In vitro [166]
	Doxorubicin (with curcumin)	Enhanced DOX efficacy via caspase activation, P-glycoprotein inhibition, and increased intracellular DOX	In vitro [167]
	Cisplatin	Reversal of cancer stem cell drug resistance; enhanced chemosensitivity; induction of cell cycle arrest and apoptosis; anti-angiogenic and pro-oxidant effects	In vitro [168]
Triptolide	Doxorubicin	Sensitization to doxorubicin via ATM suppression and inhibition of DNA damage response	In vitro [169]
	Cisplatin	Enhanced cisplatin sensitivity through disruption of XRCC1/PARP1-mediated base excision repair	In vitro [140]
Berberine	Doxorubicin	Enhanced DOX sensitivity; induction of immunogenic cell death; inhibition of cancer stem cells and oncogenic regulators (Nanog, miRNA-21)	In vivo + In vitro [170]
	Cisplatin	Induction of DNA damage and caspase-3-mediated apoptosis	In vitro [171]
Camptothecin	Doxorubicin	Reciprocal sensitization and synergistic tumor growth suppression	In vivo + In vitro [172]

Abbreviations: PI3K/AKT, phosphoinositide 3-kinase/protein kinase B; MAPK, mitogen-activated protein kinase; mTOR, mechanistic target of rapamycin; DOX, doxorubicin; ROS, reactive oxygen species; ATM, ataxia telangiectasia mutated; XRCC1, X-ray repair cross-complementing protein 1; PARP1, poly(ADP-ribose) polymerase 1; MDR1, multidrug resistance protein 1; MRP1, multidrug resistance-associated protein 1; ICAM-1, intercellular adhesion molecule 1; γH2AX, phosphorylated histone H2AX, a marker of DNA double-strand breaks. Symbols ↑ indicates upregulation or increase; ↓ indicates downregulation or decrease.

## Data Availability

No new data were created or analyzed in this study. Data sharing is not applicable to this article.

## Abbreviations

ABC	ATP-Binding Cassette
ABCG2	ATP-Binding Cassette Subfamily G Member 2
ACC	Acetyl-CoA Carboxylase
ACAT-1	Acyl-CoA:Cholesterol Acyltransferase 1
AKT	Protein Kinase B
AKT3	RAC-gamma Serine/Threonine-Protein Kinase
ALDH	Aldehyde Dehydrogenase
ALDH1A1	Aldehyde dehydrogenase 1 family member A1
AP-1	Activator Protein 1
AR	Androgen receptor
ASCT2	Alanine-Serine-Cysteine Transporter 2
ATM	Ataxia-Telangiectasia Mutated
ATP	Adenosine Triphosphate
AXL	AXL Receptor Tyrosine Kinase
BAD	BCL-2-Associated Death Promoter
BAK	BCL-2 Antagonist/Killer
BAX	BCL-2-Associated X Protein
BC	Breast Cancer
BBB	Blood–brain barrier
BBR	Berberine
BCL-2	B-cell Lymphoma 2
BCL-XL	B-cell Lymphoma Extra Large
BDNF	Brain-Derived Neurotrophic Factor
BH	BCL-2 Homology
BH3	BCL-2 Homology 3 Domain
BHG	Brain Histology Group
BHM	BCL-2 Homology Motif
BID	BH3 Interacting Domain Death Agonist
BIM	BCL-2 Interacting Mediator of Cell Death
BMI	Body Mass Index
BRCA	Breast Cancer Susceptibility Gene
CCL5	C-C Motif Chemokine Ligand 5
Caspase-9	Cysteine Aspartate Protease 9
CD3	Cluster of differentiation 3
CD4	Cluster of differentiation 4
CD8	Cluster of differentiation 8
CD8^+^ T cells	Cluster of Differentiation 8 Positive T Cells
cDC1	Conventional Dendritic Cell Type 1
CK14	Cytokeratin 14
CK5/6	Cytokeratin 5/6
CO_2_	Carbon Dioxide
COX-2	Cyclooxygenase 2
CPT	Camptothecin
CSC	Cancer Stem Cell
CSCs	Cancer Stem Cells
ctDNA	Circulating tumor DNA
CTCs	Circulating tumor cells
CXCL10	C-X-C Motif Chemokine Ligand 10
DCs	Dendritic Cells
DCIS	Ductal carcinoma in situ
ddPCR	Droplet digital polymerase chain reaction
DOX	Doxorubicin
ECM	Extracellular Matrix
EGCG	Epigallocatechin-3-Gallate
EGFR	Epidermal Growth Factor Receptor
EMT	Epithelial–Mesenchymal Transition
ER	Estrogen Receptor
ERα	Estrogen Receptor Alpha
ERK1/2	Extracellular Signal-Regulated Kinases ½
EVs	Extracellular vesicles
FA	Fatty Acid
FA-CoA	Fatty Acyl-Coenzyme A
FABPs	Fatty Acid Binding Proteins
FAD	Flavin Adenine Dinucleotide
FADH_2_	Reduced Flavin Adenine Dinucleotide
FAO	Fatty Acid Oxidation
FASN	Fatty Acid Synthase
FATPs	Fatty Acid Transport Proteins
FGFs	Fibroblast Growth Factors
FOXA1	Forkhead Box A1
GPX4	Glutathione Peroxidase 4
GLOBOCAN	Global Cancer Observatory
GLUT1	Glucose Transporter 1
GSK3β	Glycogen Synthase Kinase 3 Beta
H_2_O	Water
HDACi	Histone Deacetylase Inhibitor
HDI	Histone Deacetylase Inhibitor
HGF	Hepatocyte Growth Factor
HER2	Human Epidermal Growth Factor Receptor 2
HIF	Hypoxia-Inducible Factor
HIF-1α	Hypoxia-Inducible Factor 1 Alpha
HIF-2α	Hypoxia-Inducible Factor 2 Alpha
ICAM-1	Intercellular Adhesion Molecule 1
IFNγ	Interferon Gamma
IGF2	Insulin-Like Growth Factor 2
IKK	IκB Kinase
IL	Interleukin
IL-1β	Interleukin 1 Beta
IL-6	Interleukin 6
IL-12	Interleukin 12
IRF1	Interferon Regulatory Factor 1
IκBα	Inhibitor of Kappa B Alpha
JAK	Janus Kinase
JNK	c-Jun N-terminal Kinase
Ki-67	Marker of proliferation Ki-67
LAT1	L-Type Amino Acid Transporter 1
lncRNA	Long non-coding RNA
MAPK	Mitogen-Activated Protein Kinase
MCL-1	Myeloid Cell Leukemia 1
MCT1	Monocarboxylate Transporter 1
MDR	Multidrug Resistance
MDR1	Multidrug Resistance Protein 1
MET	Mesenchymal–Epithelial Transition Factor
miR-16	MicroRNA 16
miR-145	MicroRNA 145
miR-146a	MicroRNA 146a
miR-20a	MicroRNA 20a
miR-92a	MicroRNA 92a
miRNA	MicroRNA
MMP-9	Matrix Metalloproteinase 9
MMPs	Matrix Metalloproteinases
MRD	Molecular residual disease
MRI	Magnetic resonance imaging
mRNA	Messenger RNA
MRP1	Multidrug Resistance-Associated Protein 1
MT1-MMP	Membrane-Type 1 Matrix Metalloproteinase
mTOR	Mechanistic Target of Rapamycin
mTORC1	Mechanistic Target of Rapamycin Complex 1
mTORC2	Mechanistic Target of Rapamycin Complex 2
MYC	Myelocytomatosis Oncogene
NAD	Nicotinamide Adenine Dinucleotide
NADH	Reduced Nicotinamide Adenine Dinucleotide
N-cadherin	Neural Cadherin
NDDS	Nanoparticle Drug Delivery System
NF-κB	Nuclear Factor Kappa B
NGS	Next-generation sequencing
NK	Natural Killer
NLRP12	NLR Family Pyrin Domain Containing 12
NO	Nitric Oxide
NPs	Nanoparticles
NRF2	Nuclear Factor Erythroid 2–Related Factor 2
Notch1	Neurogenic Locus Notch Homolog Protein 1
OXPHOS	Oxidative Phosphorylation
PALB2	Partner and localizer of BRCA2
PARP	Poly (ADP-Ribose) Polymerase
PARP1	Poly (ADP-Ribose) Polymerase 1
PCR	Polymerase chain reaction
PD-1	Programmed Cell Death Protein 1
PD-L1	Programmed Death-Ligand 1
PGK1	Phosphoglycerate Kinase 1
PI3K	Phosphoinositide 3-Kinase
PIK3CA	Phosphatidylinositol-4,5-Bisphosphate 3-Kinase Catalytic Subunit Alpha
PIK3R1	Phosphoinositide-3-Kinase Regulatory Subunit 1
PIP3	Phosphatidylinositol (3,4,5)-Triphosphate
PKC	Protein Kinase C
PR	Progesterone Receptor
p-AKT	Phosphorylated AKT
p-ERK1/2	Phosphorylated ERK1/2
p-FAK	Phosphorylated Focal Adhesion Kinase
PTEN	Phosphatase and Tensin Homolog
PTPN12	Protein Tyrosine Phosphatase Non-Receptor Type 12
PTGS2	Prostaglandin-Endoperoxide Synthase 2
PTX	Paclitaxel
PUMA	p53 Upregulated Modulator of Apoptosis
RAD51	RAD51 recombinase
RAS	Rat Sarcoma
RIPK2	Receptor-Interacting Serine/Threonine-Protein Kinase 2
ROS	Reactive Oxygen Species
SCD	Stearoyl-CoA Desaturase
SFA	Saturated fatty acid
Slug	Snail family transcriptional repressor 2
SNAI1	Snail Family Transcriptional Repressor 1
SOD	Superoxide Dismutase
Sox-2	SRY (Sex Determining Region Y)-Box 2
STAT3	Signal Transducer and Activator of Transcription 3
TAZ	Transcriptional Coactivator with PDZ-Binding Motif
TG	Triglyceride
TGF-β	Transforming Growth Factor Beta
TGFβ-1	Transforming Growth Factor Beta 1
TILs	Tumor-infiltrating lymphocytes
TME	Tumor Microenvironment
TNBC	Triple-Negative Breast Cancer
TNF-α	Tumor Necrosis Factor Alpha
TP53	Tumor Protein P53
UFAs	Unsaturated fatty acids
uPA	Urokinase-Type Plasminogen Activator
USP8	Ubiquitin-Specific Protease 8
VE-cadherin	Vascular Endothelial Cadherin
VCAM1	Vascular Cell Adhesion Molecule 1
VEGF	Vascular Endothelial Growth Factor
VEGF-A	Vascular Endothelial Growth Factor A
VEGFA	Vascular Endothelial Growth Factor A
VEGFR	Vascular Endothelial Growth Factor Receptor
VEGFR2	Vascular Endothelial Growth Factor Receptor 2
Wnt	Wingless-Type MMTV Integration Site Family
XCL1	X-C Motif Chemokine Ligand 1
XRCC1	X-Ray Repair Cross-Complementing Protein 1
YAP	Yes-Associated Protein
ZEB1	Zinc Finger E-Box Binding Homeobox 1
ZEB2	Zinc Finger E-Box Binding Homeobox 2
β-catenin	Beta-Catenin
β-TrCP	Beta-Transducin Repeat-Containing Protein
γH2AX	Gamma H2A Histone Family Member X
